# Supplementary material for: Developing digital contact tracing tailored to haulage in East Africa to support COVID-19 surveillance: a protocol
Source: BMJ Open. 2022 Sep 2;12(9):e058457. doi: 10.1136/bmjopen-2021-058457 (PMC9441735; doi:10.1136/bmjopen-2021-058457)
Supplement: Author's manuscript [file bmjopen-2021-058457.draft_revisions.pdf]

BMJ Open

BMJ Open is committed to open peer review. As part of this commitment we make the peer review history of every article we publish publicly available.

When an article is published we post the peer reviewers' comments and the authors' responses online. We also post the versions of the paper that were used during peer review. These are the versions that the peer review comments apply to.

The versions of the paper that follow are the versions that were submitted during the peer review process. They are not the versions of record or the final published versions. They should not be cited or distributed as the published version of this manuscript.

BMJ Open is an open access journal and the full, final, typeset and author-corrected version of record of the manuscript is available on our site with no access controls, subscription charges or pay-per-view fees (http://bmjopen.bmj.com).

If you have any questions on BMJ Open's open peer review process please email info.bmjopen@bmj.com

BMJ Open

A protocol for developing digital contact tracing tailored to haulage in East Africa to support COVID-19 surveillance

Journal:	*BMJ Open*
Manuscript ID	bmjopen-2021-058457
Article Type:	Protocol
Date Submitted by the Author:	18-Oct-2021
Complete List of Authors:	Muwonge, Adrian; The University of Edinburgh The Roslin Institute, The Roslin Institute<br>Mpyangu, Christine; Makerere University College of Humanities and Social Sciences<br>Nsangi, Allen; Makerere University College of Health Sciences, Department of Medicine; Universitetet i Oslo, Institute of Health and Society, Faculty of Medicine<br>Mugerwa, Ibrahim; Ministry of Health, National Health Laboratories and Diagnostic Services, Antimicrobial Resistance National Coordination Centre (AMR-NCC) for<br>Bronsvoort, Barend M. deC.; The University of Edinburgh The Roslin Institute<br>Porphyre, Thibaud; VetAgro Sup, 89 Avenue de l'Europe, 63370 Lempdes<br>Ssebaggala, Emmanuel; Bodastage solution Limited<br>Kiayias, Aggelos; The University of Edinburgh School of Informatics, Blockchain Technology Laboratory<br>Mwaka, Erisa; Makerere University College of Health Sciences, School of Medicine<br>Joloba, Moses; Makerere University College of Health Sciences
Keywords:	COVID-19, Epidemiology < TROPICAL MEDICINE, Anthropology < TROPICAL MEDICINE

SCHOLARONE™
Manuscripts

A protocol for developing digital contact tracing tailored to haulage in East Africa to support COVID-19 surveillance

Adrian Muwonge[1]#, Christine Mpyangu[2], Allen Nsangi[3], Ibrahim Mugerwa[4], Barend M. deC. Bronsvoort[1], Thibaud Porphyre[1,5], Emmanuel Robert Ssebaggala[6], Aggelos Kiayias[1], Erisa Mwaka[3], Moses Joloba[3]

University of Edinburgh, Scotland, United Kingdom[1], Makerere University College of Humanities and Social Sciences, Kampala, Uganda[2], Makerere University College of Health Sciences, Kampala, Uganda[3], Ministry of Health, Kampala, Uganda[4], VetAgro Sup, Lyon, France[5], Bodastage Solutions, Kampala, Uganda[6],

Corresponding Author:
Dr. Adrian Muwonge
Chancellor's Fellow (Digital One-Health)
University of Edinburgh, (U.K)
Address: The Roslin Institute, Easter Bush Campus, University of Edinburgh, Midlothian, EH25 9RG, United Kingdom)
Telephone: (01316519155)
Email: Adrian.muwonge@roslin.ed.ac.uk

For peer review only

Abstract

Introduction: At the peak of Uganda's first wave of SARS-Cov2 in May 2020, one in three COVID-19 cases was linked to the haulage network. This triggered a mandatory requirement for a negative PCR test result at all ports of entry and exit (POEs), resulting in massive delays as haulage drivers were forced to wait at least 24-48 hours for their test results severely crippling the regional supply chain.

We aim to develop and test a mobile phone-based digital contact tracing (DCT) tool that could not only augment conventional epidemiological tools such as contact tracing but increase their speed and efficiency.

Methods and analysis : To test the DCT tool, we will use a stratified sample of haulage driver journeys, stratified by route type (regional and local journeys).

We will include at least 65% of the haulage driver journeys ~ 83,200 on the network through Uganda. This will allow us to capture variations in users' demographics and socio-economic statuses that could affect the use and adoption of the DCT tool. The developed DCT tool will include a mobile application and web interface to collate and intelligently process data, whose output will support decision making, resource allocation and feed mathematical models that predict epidemic waves.

The main expected result will be an open source tested Digital Contact Tracing (DCT) Tool tailored to haulage use in developing countries.

This study will inform the safe deployment of digital contact tracing technologies needed for combatting pandemics in low-income countries.

Ethics and dissemination: This work has received ethics approval from all relevant ethics committees. Locally and internationally, this work will be disseminated through peer reviewed publications, our websites https://project-thea.org/ and Github for the open source code https://github.com/project-thea/

Key words: *Digital contact-tracing, haulage, COVID-19, Uganda, East Africa*

For peer review only

Strengths and limitations of the study

Strengths

- This is the first study that fully documents the development of a DCT tool in consultation with stakeholders on the African continent.

- It brings together a multidisciplinary team to provide a solution to an old and neglected problem “the role of haulage in transmission of infectious diseases”. Therefore, although the proposed DCT tool is linked to the COVID-19 pandemic, its utility goes far beyond the current pandemic.

Weaknesses

- The ever changing epidemiological landscape of COVID-19, means the study design is subject to adjustments to accommodate the prevailing public health demands. These will however be openly highlighted and discussed in the resulting publications.

For peer review only

Background

The Corona Virus Disease of 2019 (COVID-19), caused by the SARS-CoV2 virus is a global pandemic that has stretched public health systems worldwide, often beyond carrying capacity(1, 2). The rates of transmission and rapid emergence of novel strains is particularly challenging all conventional epidemiological wisdom and by extension effective deployment of routine contact tracing tools(3). At the peak of Uganda's first COVID-19 wave, community spread was significantly attributed to haulage drivers' interacting with local communities along the road network where they would seed the infection particularly at service stations(4). This triggered an imperative requirement to demonstrate proof of health; a negative COVID-19 PCR test result for all haulage drivers at all of Uganda's ports of entry and exit (POEs) before proceeding to their destination. This however, inevitably resulted in a pile-up that brought the entire regional supply chain to a halt. For example, in May 2020, Uganda had a queue of up to 47km of haulage trucks at Busia and Malaba border posts (Uganda-Kenya border)(5), which prompted the COVID-19 task force at the Ministry of Health, in consultation with the National Logistic Platform (de facto trade union for haulage drivers) to develop a mitigation plan to this problem(**Fig 1**).

This plan aims at limiting the risk of haulage introducing the virus (and its emerging variants) into communities along the road network. It allows a haulage driver to travel for 14 days with a negative diagnostic test, therefore a faster contact tracing approach for cases and their immediate contacts is needed to inform decision making and resource allocation (6)(**Fig 1**).

Critically, effective and timely contact tracing slows the progression of community spread which addresses the economic and public health concerns that arise from mandatory lock downs(7). In this regard, case investigation and faster contact tracing are considered primary mitigating interventions to control and prevent person-to-person spread of COVID-19, as this identifies and notifies persons with a confirmed diagnosis along with their primary contacts.

Uganda has over the years developed contact tracing strategies for Ebola, Murburg and Crimean-Congo Haemorrhagic fever (CCHF) outbreaks(8). With such strategies, the surveillance teams were able to identify and isolate cases, in addition to their primary and secondary contacts. However, this "shoe string epidemiology" is too slow for fast evolving epidemics and pandemics such as COVID-19.

To improve the speed and efficiency of conventional contact tracing (CCT), geolocation features integrated in mobile phone technology have been used to track proximity which can translate into contact once one becomes a case for a disease of interest(9). This digital contact tracing (DCT) approach automatically notifies individuals who test positive and their immediate contacts to test and/or isolate. It is worth noting population-wide uptake of such technology is incumbent on the level of smart phone ownership, possibly the current bottleneck for low-income countries like Uganda. Therefore targeted use becomes the most viable alternative, however, such technology is inherently public facing as it harnesses information directly from mobile phones (10, 11). This inevitably comes with ethical, legal and sociological issues that must be robustly addressed to ensure sustainable adoption for public health. In a nutshell, DCT deployment requires a fine balance between public health benefit, which is fairly distributed against the risk of harm and basic principles of ethics (12, 13).

In countries like the United States, various forms of this technology have been used to support contact tracing with varying levels of success. Although success depends on the scale of deployment, better outcomes have been reported with targeted use for private companies particularly in Silicon Valley as well as sports franchise like American football(14, 15). Therefore countries intending to adopt this technology ought to examine and respond to the

For peer review only - http://bmjopen.bmj.com/site/about/guidelines.xhtml

following evaluation questions; a) Can a DCT tool be effective in augmenting the current public heath responses? b) If so, to what degree? c) Which specific aspects? d) With what confidence? and e) What are the prerequisites that ensure a DCT tool delivers on public health goals in a way that is ethically, legally and socially defensible?(16).

Here we target the haulage sector with an aim of showing how DCT can be developed and deployed to support public health needs in a way that meets the recommended sociological, ethical and legal boundaries in East Africa (17).

We have started by developing a DCT tool prototype, with input from the relevant stakeholders, this will be further developed and tested in Uganda. (Link to download the current prototype; https://www.project-thea.org).

Feedback will be collected through stakeholder meetings to be held throughout the project lifecycle (***Fig 2)***.

Our team will examine the interaction between end users' (knowledge, attitudes, and practices) and societal context (bioethics and legal) with regard to COVID-19 and how this influences technology uptake, and ultimately impact COVID-19 mitigation and infection control measures.

We target the haulage sector based on the hypothesis that their unique but predictable mobility patterns can be harnessed through mobile phone technology that uses: a) road network infrastructure, b) time stamped geo-positioning and c) COVID-19 test result data with the aim of increasing the speed and accuracy of public health contact tracing infrastructure.

This will be achieved through the following specific objectives:

1. To conduct stakeholder consultative meetings that inform the design of DCT tailored to the haulage sector in Uganda.
2. To develop the DCT technology to support COVID-19 surveillance.
3. To test the utility of the DCT among truck drivers
4. To develop models for mapping risk, inter transmission and estimate a haulage attribution to the national disease epidemiology profile.
5. To conduct stakeholder dialogues to explore potential benefits and harms of DCT technology on the haulage sector in general and its' impact on disease control and preparedness.

Methods

Study Design

The COVID-19 pandemic presents a continuously changing epidemiological landscape, therefore the study designs need to be adaptable to accommodate any unforeseen and unusually unpredictable circumstances. In general, the project will use a mixture of methods tailored to each of the project components. For example; i) Stakeholder engagement will use qualitative and quantitative approaches to generate data. This will be done on a representative number along the hierarchy of the stakeholders in a cross-sectional study, ii) Develop a DCT tool with stakeholder feedback for contextualisation, iii) A longitudinal study to test the DCT tool with truck drivers will capture data using the mobile application.

Study setting

For peer review only

The study will primarily focus on the East African haulage network, in particular that which transits through Uganda (***Fig 3***). Uganda has 52 ports of entry and exit (POEs), with varying levels of haulage traffic, categorised as; i) high, ii) moderate, iii) low-traffic POEs.

This study will focus on one seclusion point in Kampala and five POEs, with the heaviest traffic routes with a combined haulage network traffic coverage of 45%.

Study participants

Identification and recruitment of key stakeholders- The key stakeholders for this project are local and regional haulage truck drivers. Other stakeholders include public health experts including port-health and surveillance departments of Ministry of Health, the National Logistics Platform (NLP), Ministry of Transport, border point entry clearance and exit officials, legal, internet security and software developers, bioethicists as well as local leaders within neighbouring communities of service stations. There are approximately 123,000 registered haulage truck drivers in Uganda, 15% of whom are involved in regional haulage(18). Study participants will be recruited with guidance from the National Logistics Platform (NLP).

Sample size

Qualitative study: We will aim to recruit a diverse sample of truck drivers from any of the six study sites (**Fig 3**). Other stakeholders will be purposively selected to ensure a diverse range of experience and expertise. We estimate that a maximum of 30 participants will participate in three Focus Group Discussions (FGDs), with another 6-12 individuals taking part in the Key Informant Interviews (KII), while the self-administered questionnaire based survey will generate data from ~300 respondents. The sample size estimation for examining Knowledge attitudes and practices related to COVID-19 among haulage drivers and allied stakeholders will be done based on methodology published by this group (16)

Quantitative study: We shall conduct a survey regarding stakeholder input on the utility, functionality and privacy of the mobile application. We will aim to recruit about 65% of the ~123,000 truck drivers to test the utility of the mobile application. This estimate is based on data from countries where this type of technology has been successfully tested, with a recommendation to target approximately 60-80% of the study population (18-20). If we target 65% of the study population, this will represent 11,200 (regional haulage drivers) and 72,000 (local haulage truck drivers) respectively(21). Our inclusion criteria will be a) consent to participate in the study and b), ownership of a smart phone that runs on Android or MAC IOS operating systems.

Procedures

Objective one aims to conduct stakeholder consultative meetings to inform the design of the DCT tool tailored to the haulage sector in Uganda. In order to develop technology that embodies the values of the users, we shall conduct three stakeholder consultative meetings over the next 18 months. Using a stratified sampling method, we will identify a representative sample of key stakeholders such as regional and local haulage truck drivers, conductors and border point entry and exit clearance officials with varied characteristics that might modify the effects of the tool. These will include the individuals' age, experience, gender and education (***Fig 2***). It is noteworthy that we shall limit face to face interactions and use digital communication as much as possible, including hosting virtual structured discussions at regional and local entry and exit ports; and project meetings between partners.

For peer review only

Patient and public involvement

In addition to engaging key stakeholders as described above, we will engage research participants in the development and testing of a mobile-based digital contact tracing (DCT) tool that incorporates their in-put for contextualisation.

The first workshop will focus on; a) mapping the knowledge, attitudes and practices (KAP) around COVID-19 among stakeholders in the haulage industry. This will be done using key informant interviews (KII), structured questionnaires, as well as break-away focus group discussions (FGD). b) In addition, we shall assemble a panel of communication experts to develop awareness and sensitization materials targeting the study population. We will focus on empowering participants with the knowledge needed to protect their ethical, social and legal boundaries with regards to technology, c) We shall also conduct a survey to collect stakeholder input on the functionality and privacy settings of our mobile application.

The second workshop will focus on informed consent, for example, how one voluntarily joins or withdraws from the study, optimise parameters suitable to protect personal freedoms and importantly highlight in-built ethical and technology boundaries. It is noteworthy that the KAP survey will be done at all consultative meetings. The second focus group discussion will comprise of long-distance truck drivers, while the third will comprise among others, public health experts in order to maximise feedback on the functionality and utility of the mobile application. Approximately 30 participants will account for saturation with regard to focus group discussions (22), although each meeting will include only 10 participants at a time lasting not more than an hour.

Objective two will be to develop a DCT tool to support COVID-19 surveillance. This process uses feedback from *objective one;* to inform the development of the DCT tool. We will employ a human centred design approach- an iterative process of idea generation, prototyping, user-testing and piloting that involves end- users in the development process(23) ***(Fig 4*)**(24**)**. The application will be developed to embody values that maximise user anonymity, flexibility, minimal data storage and battery storage.

Objective three will be to test the utility of the DCT tool among haulage truck drivers. This will be achieved through truck drivers' interaction with the tool. Once the mobile application is downloaded and installed by the driver, it will; a) ask for consent from the driver to access the GPS, b) request for information on regular haulage routes such as departure and destination. In this regard, the mobile app will have a feature that a user can choose to allow its activation whenever movement relative to the road network is detected i.e. "auto activation when on road network". Finally, c) the application will then generate a universally unique identifier (UUID) or "digital token" for anonymity and deposit it into the secure centralized database. Once processes (1) to (2) in (**Fig 5)** are completed, the app will launch and run in the background.

As an option, the app will also request the user for their metadata (age, sex, district of residence and phone contact), which will also be stored encrypted on the phone. The three pieces of information i.e. UUID, sampled GPS and metadata are kept encrypted separately as an extra-data security step. It is noteworthy that the regional haulage truck drivers will be required to have a negative test prior to departure for their journey, which they do approximately 2-4 times a month (17).

COVID-19 testing: COVID-19 testing and reporting at POEs as shown by step 3 to 5 in (**Fig 5)**, will be conducted by public health officials, and a "digital token" exchange made. The mobile application will scan a QR-code of the East Africa truck driver number as their sample is collected for testing at any of the six POEs. The results along with the metadata will then be

For peer review only

posted by the laboratory into the centralised results dispatch system (RDS) managed by the Ministry of Health. The scanned QR-codes will generate a list of ID's which our system will use to retrieve results from the RDS. Here the DCT system will do the following; a) retrieve and notify the driver of their results, b) if the result is positive, the driver will receive a notification and instructions on what to do to protect the public, c) The tracking data will then be used to create 7 day risk map for cases which will be available as web interface accessible to the public health surveillance team to support decision making. We believe that when fully functional, the system can also inform risk-based screening which would save haulage companies, tens of thousands of US Dollars in testing.

Contact tracing: This will be conducted via the web interface only accessible to the Ministry of Health. The interface will generate a contact graph from the time-stamped GPS locations relative to the road infrastructure Step 5, in (**Fig 5)**. It will then be overlaid with the COVID test results to reveal the location and trail of the cases. At this point, the metadata (age, sex, district of residence and phone contact) collected by public health officials at the testing stations can be used to directly contact the individual as a follow up to the mobile application-based alert. Then, risk assessment i.e. using time-stamped geodata to establish who has been at the same stops along the network and for how long). This is then used to generate a list of at-risk drivers who will be automatically alerted through the mobile app. The option here could be for the mobile app to avail mobile phone numbers sampled from metadata to follow-up on the mobile app alert.

Objective four will be to develop models for mapping risk, infer transmission and estimate a haulage attribution - The data collected by the mobile application (DCT) will be analysed as follows: Firstly, we will use machine learning methods to compute the probability of drivers testing positive as a function of characteristics of their movements (e.g. their route, duration of the journey, the departure time, vehicle type (~fuelling frequency) estimated traffic and contact structure. Determining a probabilistic output will enable the public health officials to prioritise who to test (risk-based diagnostics), hence limiting the amount of contacts between infected drivers and individuals of the community. Note that since this process will need a training dataset, the first four months will be to generate this data set (~ 160,000 trips). Therefore the utility of this approach for this analysis will be after five months of data collection.

Secondly the movements of trucks along the road networks will form a large network where nodes such as villages/towns/cities and edges are movements of haulage vehicles. This network will then be analysed to evaluate which communities are key in the structure of the network, their potential for disease transmission and infection as well as their potential as candidates for placing efficient testing facility outposts.

Lastly, assuming all drivers test negative at departure, we will estimate the likelihood of infection from communities along each individual travel route based on the spatial and temporal aspect of each individual journey, the time of testing, factoring in the epidemiological characteristics of COVID-19 (latent, incubation and symptomatic periods) in addition to probability of clinical signs. Based on this evidence, communities with a high risk of infection (either as source or recipient of infection) will be identified. Such a risk assessment would provide timely evidence for targeting communities with high risk of disease and improve resource allocations. By combining this with other data, we shall estimate the contribution of haulage to the epidemic and evaluate the impact of using this intervention on COVID-19 control, however the last component of the analysis will likely occur beyond the 18 months project period.

Objective five is to conduct stakeholder dialogues to explore the impact of the mobile application (DCT) on disease control and preparedness. Deliberative dialogues are a form of public discourse that are currently used to develop a shared understanding of a problem or

For peer review only

solution among key stakeholders(25). The objectives of these dialogues will "not so much to talk together as to think together, not so much to reach a conclusion as to discover where a conclusion might lie" and to ensure that different viewpoints and assumptions are explored(26). Dialogues also provide opportunities for tacit knowledge to be shared(27, 28).Using deliberative dialogues, we will create spaces where study findings are discussed and explored by key stakeholders, provide opportunities to examine the extent to which the study findings are applicable to their contexts and how use of the mobile application technology could be formalized and scaled up to ensure appropriate governance and mechanisms to maintain data privacy. These stakeholder dialogues will follow the same approach as described for the KAP above.

Qualitative data analysis- will be done using interpretative description approaches (29), such as those used in ethnography and naturalistic inquiry to analyse data arising from the KAP survey, KII and FGDs. These approaches allow us to examine the practical implications of DCT on the routine activities of the truck drivers in the context of the existing body of knowledge in an iterative analysis, while exploring challenges and opportunities to using DCT technologies. We shall use qualitative analysis software, such as Nvivo to code and interrogate our data.

Data collection and management

The contact tracing component will primarily use the collected time-stamped GPS data, which data will be strictly limited to the road network, securely collected and transmitted to a server. The data collected will be stored in relational database and retrievable in a number of formats including csv for analysis. The database will be replicated online to provide redundancy and ensure uninterrupted operation. In addition, daily backups are taken in the event that we need to restore the data which data will be kept for the duration of the project. Use of this data, will only be triggered by a case, it is this case's risk profile that informs the contact tracing and notification via the mobile application. Otherwise all data will be kept separate. Diagnostic data will be generated and posted to a secure public health centralised system also referred to as the Result Dispatch System (RDS).

The stakeholder engagement component will generate qualitative data, collected as part of the Knowledge Attitude and Practices analysis linked to COVID-19, ethics and legal aspects of technology deployment. It will consist of audio recordings, observational notes, transcribed texts and still photography. Participants will be required to consent before downloading or installing the app. All participant data will be anonymised and stored securely on our servers for analysis. In addition, sequence data will be generated from a selected number of samples using Oxford Nano pore, this data once superimposed on the network, allows us to monitor the routes of COVID-19 strain introduction. The processing of this data will generate secondary data to be securely stored at the African Center for Excellence in Bio Informatics (ACE) at the Infectious Diseases Institute at Makerere University.

Project deliverables

1. *Open source Digital Contact Tracing Tool (DCT):*

We shall develop an open source Digital Contact Tracing (DCT) tool tailored to haulage in Uganda, to enhance the speed and efficiency of the current manual of public health contact tracing system. In so doing, we shall redirect resources from manual tracing, subsequently reducing border transit time for haulage while indirectly supporting increased supply chain flow.

For peer review only

2. *Statistical and mathematical models:*

Furthermore, the modelling team will develop statistical and mathematical models using output from testing for epidemic prediction and risk mapping. The outputs will provide evidence to inform, a) public health decision making on personnel and resource allocation critical to improving the efficacy, cost-efficiency and ultimately safety and surveillance of contact tracing activities, b) future control strategies to mitigate the spread of COVID-19 and other subsequent novel pathogens while limiting economic consequences and strengthening the resilience of the commodities' supply chain.

3. *Avail evidence for vaccination programs:*

The contact tracing analysis will provide epidemic characteristics, such as the speed of spatial transmission and size of the epidemic in specific locations, for example current data on hotspots. The data can then be used to define prioritisation of vaccination, estimate vaccination coverage while providing evidence on vaccination impact (i.e. temporal and spatial-changes) in the epidemic size.

4. *Avail evidence for policy:*

Lastly, any lessons learned regarding the legal, ethical and social-anthropological examination related to DCT deployment will be used to regulate usage and inform policy formulation in relation to use of digital contact tracing technology in combatting pandemics.

5. Interpretation and reporting of findings

Building on the deliberative dialogues, a small group of key stakeholders will be engaged in interpreting and confirming interpretations of findings. They will be invited to comment on reports and be co-authors.

Ethical considerations

In Uganda, ethics approval was obtained from School of Public Health Higher Degrees, Research and Ethics Committee at Makerere University (approval number SPH-2021-35) and Uganda National Council for Science and Technology (approval number HS156ES). At the University of Edinburgh, study approval was granted by the Human Ethics and Research Committee at the Easter Bush Campus (HERC_538).

We will obtain informed consent for participation in interviews, focus group discussions, still photography and videography. Data from interviews, questionnaires, GPS, photos, videos and notes will be anonymised.

Benefits of participation

Since clinically valid tests such as COVID-19 PCR test, are part of this protocol, research participants may benefit from individual results provided conveniently without financial cost. However, since research participants will not be provided any therapeutic interventions, consent forms clearly state that no financial benefits shall be gained as a result of individual participation in this research study, however haulage companies may ultimately benefit monetarily by reducing their employees' exposure to the virus thereby limiting sickness-related absences while maintaining operations.

Risks of participation

In general, risks of participation resulting from both psychological and physical intervention necessary to acquire a biological sample are trivial. The primary risks to participation in this study are generally psychological, such as stigma and discrimination if sensitive information is generated in the project and there is a breach of privacy or confidentiality. However, maximum standards that ensure confidentiality and privacy will be observed and maintained at all levels of data collection and processing to further mitigate that risk. No personal

For peer review only

identification details will be collected as participants will be assigned unique identification codes, “digital tokens”. The project will be conducted in accordance with the Ministry of Health and Uganda National Council for Science and Technology guidelines for the prevention of spread of COVID- 19.

Dissemination and reporting

We will provide all participating organisations with a report of the main findings as soon as the analyses have been completed and independently verified by at least two referees who are not part of the study team.

We will actively disseminate the results of this study through publications and presentations. At National level, the study is nested within the Ministry of Heath, with study outputs directly feeding into the public health responses via incident command teams. At the regional level, study findings will be presented at regional haulage sector meetings facilitated by the National Logistics Platform in Uganda. Internationally, we will actively disseminate the study findings through peer reviewed publications, pre-existing partnerships and our organisation websites such as https://project-thea.org/ and Github account for the open source code https://github.com/project-thea/

Data availability statement

The data files resulting from this study will be made available at reasonable request.

Funding

This study is funded by the National Institute of Health Research/ Medical Research Council-GECO award, ref MR/V034952/1

Author contributions

Conceptualisation: (Adrian Muwonge, Emmanuel Ssebaggala, Moses Joloba, Ibrahim Mugerwa)
Funding acquisition: (Adrian Muwonge, Christine Mpyangu, Ibrahim Mugerwa, Mark Bronsvoort[1], Thibaud Porphyre, Emmanuel Robert Ssebaggala, Aggelos Kiayias, Erisa Mwaka, Moses Joloba)
Investigation: Adrian Muwonge, Christine Mpyangu, Allen Nsangi, Ibrahim Mugerwa, Mark Bronsvoort[1], Thibaud Porphyre, Emmanuel Robert Ssebaggala, Aggelos Kiayias, Erisa Mwaka, Moses Joloba
Writing- original draft: (Adrian Muwonge, Christine Mpyangu, Allen Nsangi, Ibrahim Mugerwa, Mark Bronsvoort[1], Thibaud Porphyre, Emmanuel Robert Ssebaggala, Aggelos Kiayias, Erisa Mwaka, Moses Joloba)
Writing- review and editing: Adrian Muwonge, Christine Mpyangu, Allen Nsangi, Ibrahim Mugerwa, Mark Bronsvoort[1], Thibaud Porphyre, Emmanuel Robert Ssebaggala, Aggelos Kiayias, Erisa Mwaka, Moses Joloba

Declaration of interests

We declare no competing interests.

For peer review only

References

1. El-Sadr W, Justman J. Africa in the path of covid-19. N Engl J Med. 2020;383:e11.
2. Tumwesigye NM, Denis O, Kaakyo M, Biribawa C. Effects of the COVID-19 Pandemic on Health Services and Mitigation Measures in Uganda. Center for Global Development, 2021.
3. World Health Organization. Global research on coronavirus disease (COVID-19). World Health Organisation. 2021.
4. Ministry of Health. Coronavirus (COVID-19) Pandemic; COVID-19 Testing File Report-August 03 2020. Ministry of Health- Uganda. 2020.
5. Journal TWS. On East Africa's Borders, Vast Jams as Truckers Are Tested for Covid-19. The Wall Street Journal. 2020.
6. Roberts SL. Big Data, Algorithmic Governmentality and the Regulation of Pandemic Risk. European Journalof Risk Regulation,. 2019;10(1):94-115.
7. Haider N, Osman AY, Gadzekpo A, Akipede GO, Asogun D, Ansumana R, et al. Lockdown measures in response to COVID-19 in nine sub-Saharan African countries. BMJ Global Health. 2020;5(10):e003319.
8. Okware S. Managing Ebola in low-resource settings: experiences from Uganda. In Ebola IntechOpen
2016.
9. Blasimme A, Ferretti A, Vayena E. Digital Contact Tracing Against COVID-19 in Europe: Current Features and Ongoing Developments. Frontiers in Digital Health. 2021;3(61).
10. Mooney G. A Menace to the Public Health"—Contact Tracing and the Limits of Persuasion. New England Journal of Medicine. 2020.
11. Kahn JP. Digital contact tracing for pandemic response: Ethics and governance guidance. . John Hopkins University Press. 2020.
12. Bull S, Et al. Best practices for ethical sharing of individual-level health research data from low-and middle-income settings. . Journal of Empirical Research on Human Research Ethics. 2015;10(3):302-13.
13. Gasser U, Et al. Digital tools against COVID-19: taxonomy, ethical challenges, and navigation aid. Lancet Digital Health. 2020.
14. Kane C. How Kinexon — a German company with U.S. headquarters in Chicago — is tracking players' daily interactions to prevent the spread of COVID-19 in the NFL. 2020.
15. Center for Disease Control and Prevention. Digital contact tracing tools. 2020.
16. Muleme J, Kankya C, Ssempebwa JC, Mazeri S, Muwonge A. A Framework for Integrating Qualitative and Quantitative Data in Knowledge, Attitude, and Practice Studies: A Case Study of Pesticide Usage in Eastern Uganda. Frontiers in Public Health. 2017;5(318).
17. Keeling MJ, Hollingswworth TD, Read JM. The Efficacy of Contact Tracing for the Containment of the 2019 Novel Coronavirus (COVID-19). . medRxiv,. 2020.
18. BraithwaiteI I, Et al. Automated and partly automated contact tracing: a systematic review to inform the control of COVID-19. Lancet Digital Health. 2020.
19. Kim MS. Seoul's radical experiment in digital contact tracing 2020.
20. Rowe F. Contact tracing apps and values dilemmas: A privacy paradox in a neo-liberal world. International Journal of Information Management. 2020;55(102178).

For peer review only

21. Eberhard-Ruiz A, Calabrese L. Trade facilitation, transport costs and the price of trucking services in East Africa. Working and discussionpapers (August 2017) 2017.
22. Guest G BA, Johnson L. How many interviews Are enough?: anexperiment with data saturation and variability. Field Meth. 2006;2006(18):5-82.
23. Abras C, Maloney-Krichmar D, Preece J. User-Centered Design. In: Bainbridge W, editor. Encyclopedia of Human-Computer Interaction. Thousand Oaks:Sage Publi-cations, 2004;. 2004.
24. IDEO.org. The Field Guide to Human-Centered Design. . San Francisco: IDEOorg,. 2015;https://www.designkit.org/resources/1
25. The SURE Collaboration. Informing and engaging stakeholders. In: SURE Guides for Preparing and Using Evidence-Based Policy Briefs. Geneva: World Health Organization,. 2011;https://www.who.int/evidence/sure/guides/en/ 2011;Version 2.1(https://www.who.int/evidence/sure/guides/en/).
26. INVOLVE. People & Participation: How to put citizens at the heart of decision-making. London: Involve. 2005;https://www.involve.org.uk/resources/publications/practical-guidance/people-and-participation.
27. Hadjimichael D, Tsoukas H. Toward a better understanding of tacit knowledge in organizations: Taking stock and moving forward. Academy of Management Annals 2019;13(2):672-703.
28. Kothari A, Rudman D, Dobbins M, Et al. The use of tacit and explicit knowledge in public health: a qualitative study. Implementation Science. 2012;7(20).
29. Thorne S, Kirkham SR, O'Flynn-Magee K. The analytic challenge in interpretive description. Int J Qual Methods. 2004;3(1-11).

Figure legend

Figure 1: The study is responding to a public health and economic **challenge** linked to haulage sector as shown above. This group represents a **risk** of introducing COVID-19 and its variants to communities along the road infrastructure such as service stations, resting points and restaurants. This risk is exacerbated by delays which cause queuing up at POEs. Our **solution** is aimed at improving the efficiency of conventional contact tracing using **digital contact tracing**. The **utility**- is that this would allow for timely and robust **risk assessment**, automatic notification of primary and secondary contacts of identified cases as well as prediction of hotspots along the haulage network to support public health preparedness. The image was generated using Biorender https://biorender.com

Figure 2: Shows the conceptual framework on which this study is developed and will be implemented. We posit that technology update is enhanced by understanding inherent characteristics of the intended user (attitudes, knowledge and how these translate into actions) and the characteristics of the community they inhabit (legal, ethical, traditions and connectivity access). The project has four components; A is stakeholder engagement and inclusion, B-Technology development, C- Testing the technology and D -Data analysis to support public health interventions. The image was generated using Biorender https://biorender.com

Figure 3: Shows the convergence of the East African haulage network in Uganda. The study will focus on the six ports of Entry and Exits (POEs) shown as circular highlights. The image was generated using Bio render https://biorender.com

For peer review only

697 **Figure 4:** Shows the iteration stages of the Human Centred Design Approach

699 **Figure 5:** Demonstrates step by step the THEA-C19 mobile application installation and
700 contact tracing processes

For peer review only

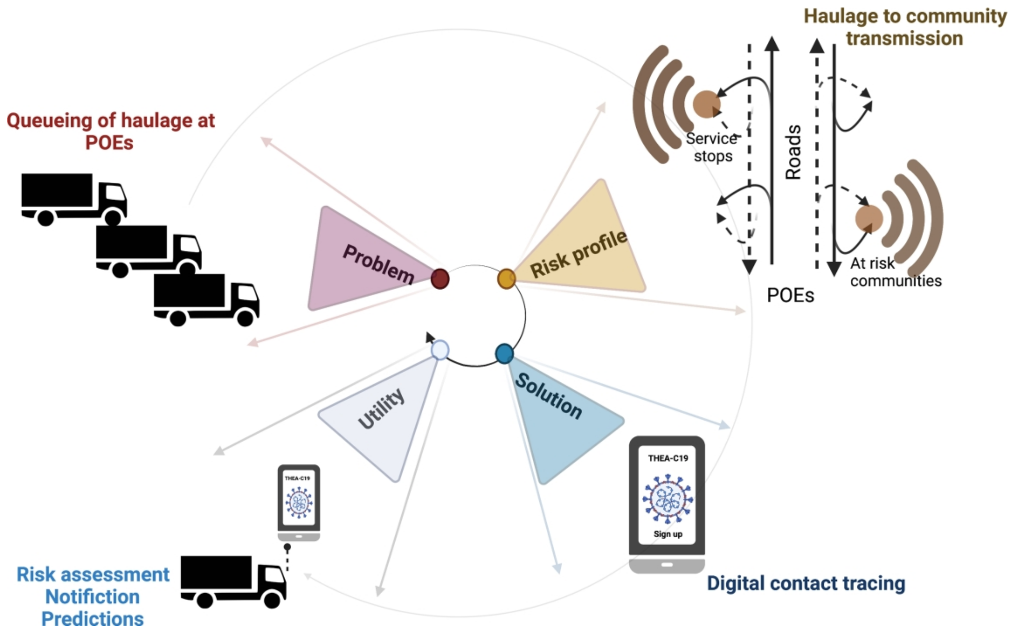

Figure 1: The study is responding to a public health and economic challenge linked to haulage sector as shown above. This group represents a risk of introducing COVID-19 and its variants to communities along the road infrastructure such as service stations, resting points and restaurants. This risk is exacerbated by delays which cause queuing up at POEs. Our solution is aimed at improving the efficiency of conventional contact tracing using digital contact tracing. The utility- is that this would allow for timely and robust risk assessment, automatic notification of primary and secondary contacts of identified cases as well as prediction of hotspots along the haulage network to support public health preparedness. The image was generated using Biorender https://biorender.com

249x154mm (300 x 300 DPI)

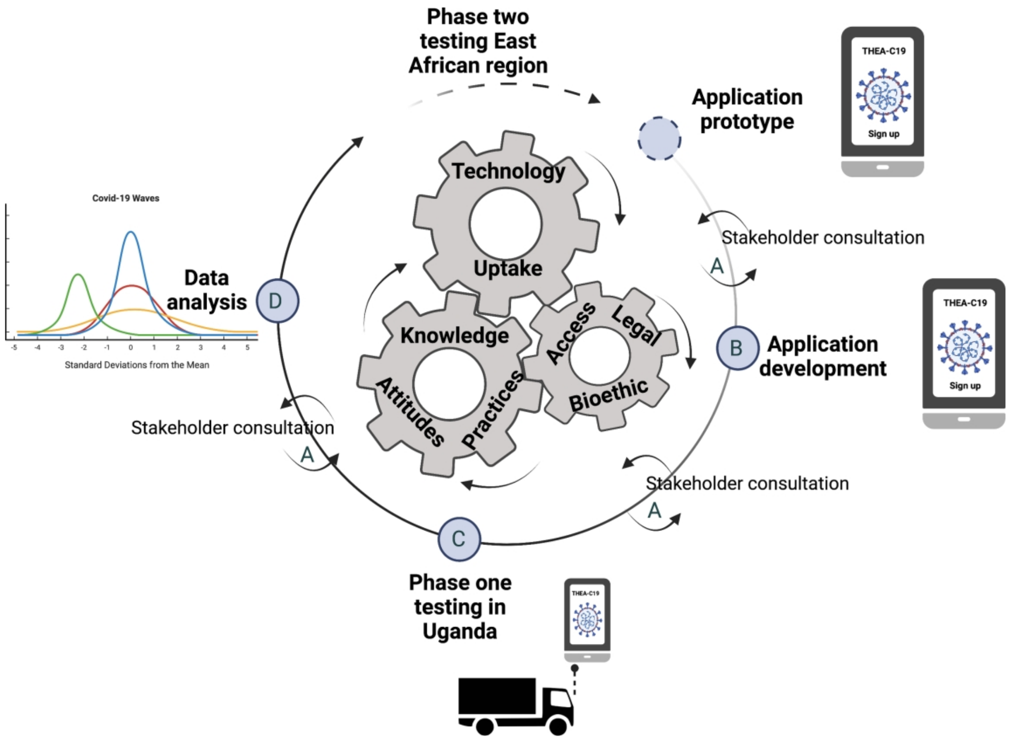

Figure 2: Shows the conceptual framework on which this study is developed and will be implemented. We posit that technology update is enhanced by understanding inherent characteristics of the intended user (attitudes, knowledge and how these translate into actions) and the characteristics of the community they inhabit (legal, ethical, traditions and connectivity access). The project has four components; A is stakeholder engagement and inclusion, B- Technology development, C- Testing the technology and D -Data analysis to support public health interventions. The image was generated using Biorender https://biorender.com

229x166mm (300 x 300 DPI)

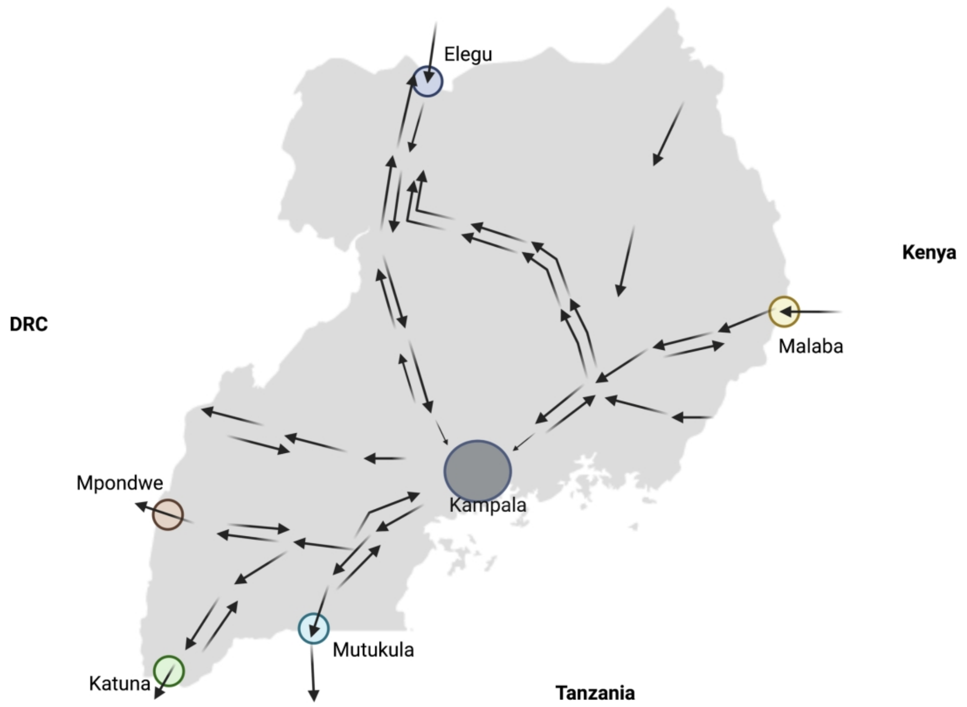

Figure 3: Shows the convergence of the East African haulage network in Uganda. The study will focus on the six ports of Entry and Exits (POEs) shown as circular highlights. The image was generated using Bio render https://biorender.com

253x177mm (300 x 300 DPI)

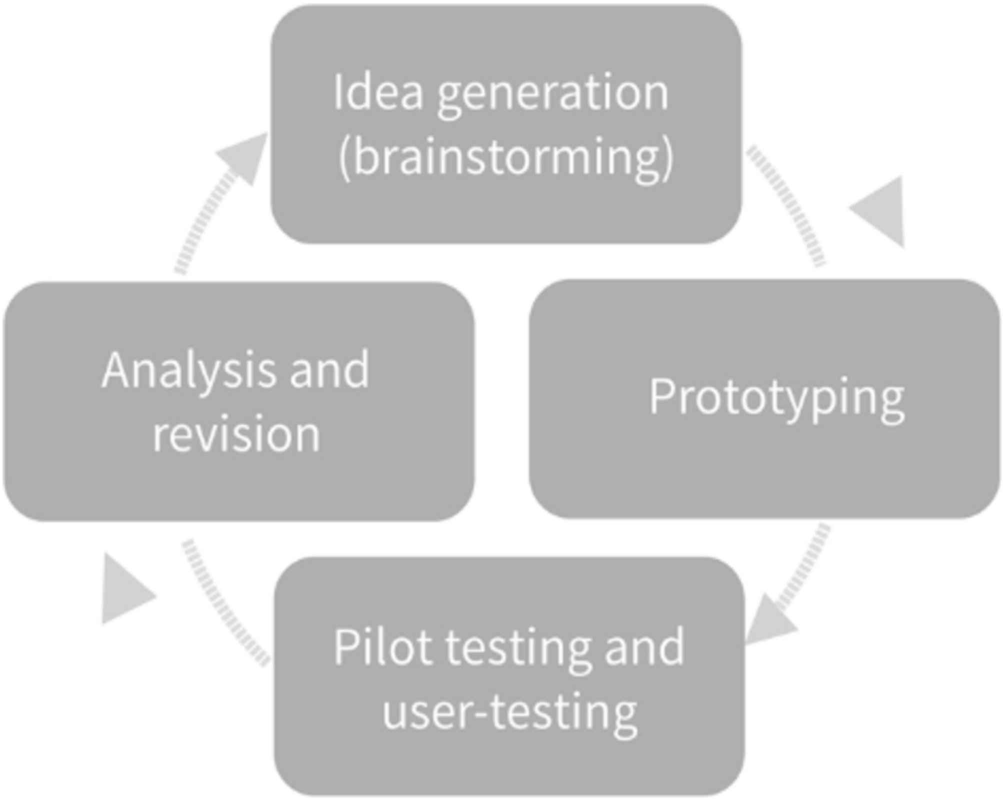

Figure 4: Shows the iteration stages of the Human Centred Design Approach

190x151mm (300 x 300 DPI)

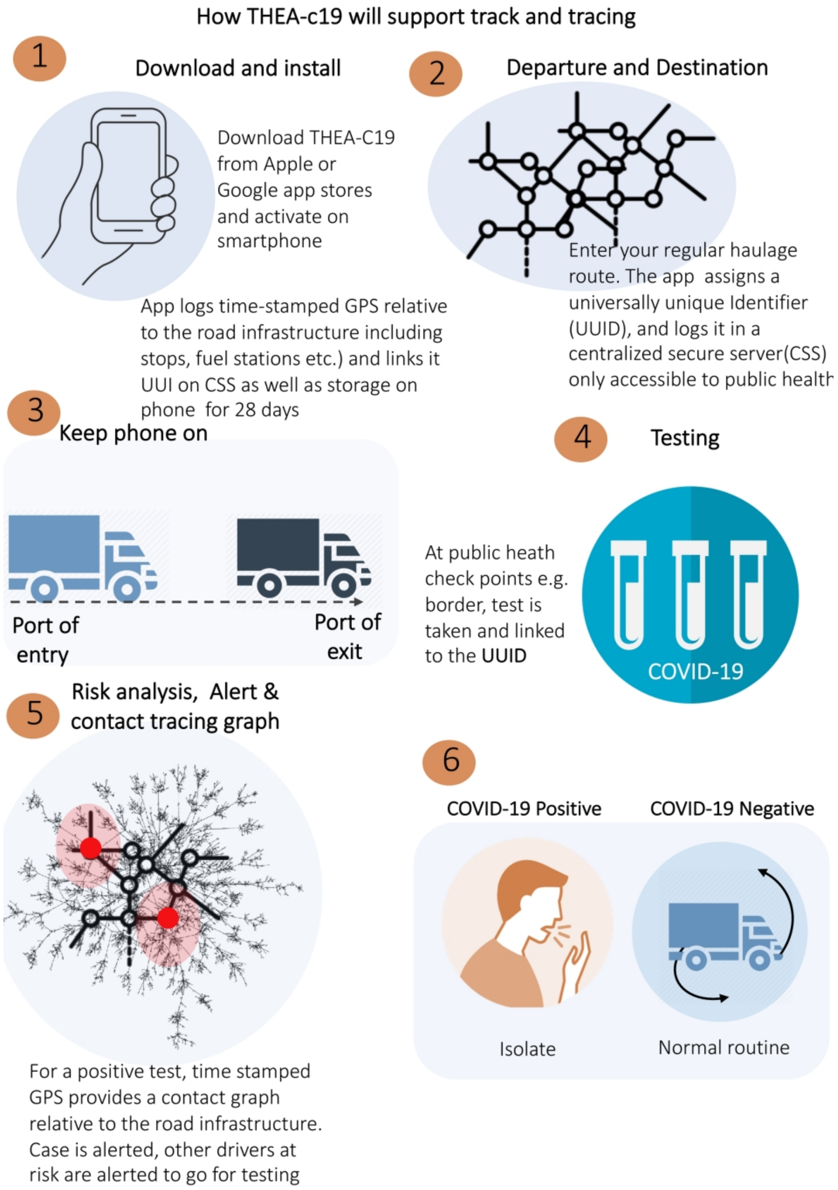

Figure 5: Demonstrates step by step the THEA-C19 mobile application installation and contact tracing processes

190x275mm (300 x 300 DPI)

THE ROSLIN INSTITUTE
The University of Edinburgh
Easter Bush
Midlothian
EH25 9RG
Telephone: +44 (0)131 651 9100
www.roslin.ed.ac.uk

Dear Editor
BMJ protocol

07.10.2021

Re: A protocol for developing digital contact tracing tailored to haulage in East Africa to support COVID-19 surveillance

I am delighted to finally submit this manuscript for consideration in BMJ protocols. It represents one year's worth of work since this NIHR/MRC GECO award was made. These awards were made to support critical aspects of the SARS-COVID-19 pandemic in various parts of the developing world.

In Uganda for example; one in three COVID-19 cases during the first wave (May 2020) was a haulage truck driver. This presented a unique challenge for this landlocked country at the heart of the East African haulage network. To prevent community transmission, it became mandatory to provide proof of health (a negative test result) at all 53 ports of entry and exits (POEs) in Uganda.

This resulted in long haulage truck queues at POEs, inevitably grinding the regional supply chain to a halt. As a solution, haulage drivers would be tested and allowed to continue with their journeys, but this required a fast approach for contact tracing if one tested positive. It soon became evident that conventional epidemiological contact tracing was not suitable given the speed of transmission of COVID-19. Our team then proposed to develop a digital contact tracing system tailored to this risk group. We deliberately chose to develop a targeted digital contact tracing tool to generated critical data needed to underwrite the use and regulation of such technology for public health in developing countries.

At the time, various parts of the world were testing this technology on populations with various levels of success. For example South Korea's apparent success was their ability to combine proximity data with triangulated information from government held personal datasets, so the reported success in public health came at a loss of individual freedoms.

On the other hand, where a balance between individual freedoms and public health utility was a consideration, the delivery of this technology became difficult and expensive. Interestingly, at the same time targeted use of digital contact tracing was registering success in maintaining a "safe burble" which allowed resumption of elite sports such as the American football.

These aspects highlighted fundamental gaps which necessitated investigating the development and deployment of such technology especially in developing countries. For example what are the legal, ethical and socio-anthropological boundaries deploying digital contact tracing for public health?

Answering this question is central to the sustainable use of this technology for public health. Our solution will show how such technology can be locally developed and implemented in consultation with stakeholder. In so doing, we examine and document the socio-economic, ethical, technological and legal boundaries of deploying such technology. We also provide an open source version of the tools which can be adapted for the developing part of the world. We therefore believe that publication

For peer review only - http://bmjopen.bmj.com/site/about/guidelines.xhtml

of this work in **BMJ protocol** provides impetus for further development and adoption to drive sustainable and responsible use of digital contact tracing for public health.

The submitted manuscript is slightly over the recommended word limit of 4000 words, currently at 5394 words inclusive of the abstract (290 words) and references (621 words). We request that the manuscript is granted an exception to facilitate a thorough examinations of the issues as stipulated therein.

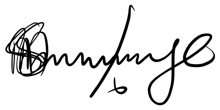

Adrian Muwonge (DVM, MPH, PhD)

Chancellor's Fellow (Digital One-health)
Deputy Director Fleming Fund Scholarships (Uganda & Malawi)
Div. Genetics and Genomics, The Roslin Institute
University of Edinburgh

For peer review only

CONSERVE Checklists

Use CONSERVE-CONSORT for completed trial reports and CONSERVE-SPIRIT for trial protocols.

CONSERVE-CONSORT Extension: [DATE]

**Item**	**Item Title**	**Description**	**Page No.**
I.	Extenuating Circumstances	Describe the circumstances and how they constitute extenuating circumstances.	
II.	Important Modifications	a. Describe how the modifications are important modifications.	
		b. Describe the impacts and mitigating strategies, including their rationale and implications for the trial.	(see below)
		c. Provide a modification timeline.	
III.	Responsible Parties	State who planned, reviewed and approved the modifications.	
IV.	Interim data	If modifications were informed by trial data, describe how the interim data were used, including whether they were examined by study group, and whether the individuals reviewing the data were blinded to the treatment allocation.	

**CONSORT Number and Item**		For each row, if important modifications occurred check "direct impact" and/or "mitigating strategy" and describe the changes in the trial manuscript or supplement. Check "no change" for items that are unaffected in the extenuating circumstance.			**Page No.**
		**No Change**	**Impact***	**Mitigating Strategy****	
1	Title and abstract				
2	Introduction				
3	Methods: Trial Design				
4	Methods: Participants				
5	Methods: Interventions				
6	Methods: Outcomes				

7	Methods: Sample Size				
8-10	Methods: Randomisation				
11	Methods: Blinding				
12	Methods: Statistical methods				
13	Results: Participant flow				
14	Results: Recruitment				
15	Results: Baseline data				
16	Results: Numbers analysed				
	Results: Outcomes and estimation				
18	Results: Ancillary analyses				
19	Results: Harms				
20	Discussion: Limitations				
21	Discussion: Generalisability				
	Other information: Registration				
24	Other information: Protocol				
25	Other information: Funding				

*Aspects of the trial that are directly affected or changed by the extenuating circumstance and are not under the control of investigators, sponsor or funder.
**Aspects of the trial that are modified by the study investigators, sponsor or funder to respond to the extenuating circumstance or manage the direct impacts on the trial.

CONSERVE-SPIRIT Extension: [15th October 2021]					
**Item**	**Item Title**	**Description**			**Page No.**
I.	Extenuating Circumstances	Describe the circumstances and how they constitute extenuating circumstances.			N/A
II.	Important Modifications	a. Describe how the modifications are important modifications.			N/A
		b. Describe the impacts and mitigating strategies, including their rationale and implications for the trial.			(see below)
		c. Provide a modification timeline.			N/A
III.	Responsible Parties	State who planned, reviewed and approved the modifications.			N/A
IV.	Interim data	If modifications were informed by trial data, describe how the interim data were used, including whether they were examined by study group, and whether the individuals reviewing the data were blinded to the treatment allocation.			N/A
**SPIRIT Item and Number**		For each row, if important modifications occurred, check one or both of "impact" and/or "mitigating strategy" and describe the changes in the protocol. Check "no change" for items that are unaffected in the extenuating circumstance.			**Page No.**
		**No Change**	**Impact***	**Mitigating Strategy****	
1	Title	√			1
2	Trial registration	√			N/A
3	Protocol version	√			N/A
4	Funding	√			11
5	Roles and responsibilities	√			11
6	Background and rationale	√			04
7	Objectives	√			05
8	Trial design	√			05
9	Study setting	√			05
10	Eligibility criteria	√			06
11	Interventions	√			05
12	Outcomes	√			09

For peer review only

13	Participant timeline	√			06
14	Sample size	√			06
15	Recruitment	√			06
16	Allocation	√			N/A
17	Blinding (masking)	√			N/A
18	Data collection methods	√			09
19	Data management	√			09
20	Statistical methods	√			09
21	Data monitoring	√			09
22	Harms	√			10
23	Auditing	√			N/A
24	Research ethics approval	√			10
25	Protocol amendments	√			N/A
26	Consent or assent	√			06
27	Confidentiality	√			10
28	Declaration of interests	√			11
29	Access to data	√			11
30	Ancillary and post-trial care	√			N/A
31	Dissemination policy	√			11
32	Informed consent materials	√			10
33	Biological specimens	√			N/A

*Aspects of the trial that are directly affected or changed by the extenuating circumstance and are not under the control of investigators, sponsor or funder.
**Aspects of the trial that are modified by the study investigators, sponsor or funder to respond to the extenuating circumstance or manage the direct impacts on the trial.

For peer review only - http://bmjopen.bmj.com/site/about/guidelines.xhtml

BMJ Open

A protocol for developing digital contact tracing tailored to haulage in East Africa to support COVID-19 surveillance

Journal:	*BMJ Open*
Manuscript ID	bmjopen-2021-058457.R1
Article Type:	Protocol
Date Submitted by the Author:	25-Feb-2022
Complete List of Authors:	Muwonge, Adrian; The University of Edinburgh The Roslin Institute, The Roslin Institute<br>Mpyangu, Christine; Makerere University College of Humanities and Social Sciences<br>Nsangi, Allen; Makerere University College of Health Sciences, Department of Medicine; Universitetet i Oslo, Institute of Health and Society, Faculty of Medicine<br>Mugerwa, Ibrahim; Ministry of Health, National Health Laboratories and Diagnostic Services, Antimicrobial Resistance National Coordination Centre (AMR-NCC) for<br>Bronsvoort, Barend M. deC.; The University of Edinburgh The Roslin Institute<br>Porphyre, Thibaud; VetAgro Sup, 89 Avenue de l'Europe, 63370 Lempdes<br>Ssebaggala, Emmanuel; Bodastage solution Limited<br>Kiayias, Aggelos; The University of Edinburgh School of Informatics, Blockchain Technology Laboratory<br>Mwaka, Erisa; Makerere University College of Health Sciences, School of Medicine<br>Joloba, Moses; Makerere University College of Health Sciences
**Primary Subject Heading**:	Public health
Secondary Subject Heading:	Epidemiology, Evidence based practice, Global health, Infectious diseases
Keywords:	COVID-19, Epidemiology < TROPICAL MEDICINE, Anthropology < TROPICAL MEDICINE, Public health < INFECTIOUS DISEASES

SCHOLARONE™
Manuscripts

A protocol for developing digital contact tracing tailored to haulage in East Africa to support COVID-19 surveillance

Adrian Muwonge[1]#, Christine Mpyangu[2], Allen Nsangi[3], Ibrahim Mugerwa[4], Barend M. deC. Bronsvoort[1], Thibaud Porphyre[1,5], Emmanuel Robert Ssebaggala[6], Aggelos Kiayias[1], Erisa Mwaka[3], Moses Joloba[3]

University of Edinburgh, Scotland, United Kingdom[1], Makerere University College of Humanities and Social Sciences, Kampala, Uganda[2], Makerere University College of Health Sciences, Kampala, Uganda[3], Ministry of Health, Kampala, Uganda[4], VetAgro Sup, Lyon, France[5], Bodastage Solutions, Kampala, Uganda[6],

Corresponding Author:
Dr. Adrian Muwonge
Chancellor's Fellow (Digital One-Health)
University of Edinburgh, (U.K)
Address: The Roslin Institute, Easter Bush Campus, University of Edinburgh, Midlothian, EH25 9RG, United Kingdom)
Telephone: (01316519155)
Email: Adrian.muwonge@roslin.ed.ac.uk

For peer review only

Abstract

Introduction: At the peak of Uganda's first wave of SARS-Cov2 in May 2020, one in three COVID-19 cases was linked to the haulage sector. This triggered a mandatory requirement for a negative PCR test result at all ports of entry and exit (POEs), resulting in significant delays as haulage drivers had to wait for 24-48 hours for results, which severely crippled the regional supply chain.

To support public health and economic recovery, we aim to develop and test a mobile phone-based digital contact tracing (DCT) tool that not only augments conventional contact tracing but increases its speed and efficiency.

Methods and analysis: To test the DCT tool, we will use a stratified sample of haulage driver journeys, stratified by route type (regional and local journeys).

We will include at least 65% of the haulage driver journeys ~ 83,200 on the network through Uganda. This allows us to capture variations in user demographics and socio-economic characteristics that could influence the use and adoption of the DCT tool. The developed DCT tool will include a mobile application and web interface to collate and intelligently process data, whose output will support decision making, resource allocation and feed mathematical models that predict epidemic waves.

The main expected result will be an open source tested Digital Contact Tracing (DCT) Tool tailored to haulage use in developing countries.

This study will inform the safe deployment of digital contact tracing technologies needed for combatting pandemics in low-income countries.

Ethics and dissemination: This work has received ethics approval from all relevant ethics committees. Locally and internationally, this work will be disseminated through peer reviewed publications, our websites https://project-thea.org/ and Github for the open source code https://github.com/project-thea/

Key words: *Digital contact-tracing, haulage, COVID-19, Uganda, East Africa*

For peer review only

Strengths and limitations of the study

Strengths

- This is the first study that fully documents the development of a DCT tool in consultation with stakeholders on the African continent.

- It brings together a multidisciplinary team to provide a solution to an old and neglected problem "the role of haulage in transmission of infectious diseases". Therefore, although the proposed DCT tool is linked to the COVID-19 pandemic, its utility goes far beyond the current pandemic.

Weaknesses

- The ever-changing epidemiological landscape of COVID-19, means the study design is subject to adjustments to accommodate the prevailing public health demands. These will however be openly highlighted and discussed in the resulting publications.

For peer review only

Background

The Corona Virus Disease of 2019 (COVID-19), caused by the SARS-CoV2 virus, is a global pandemic that has stretched public health systems worldwide, often beyond carrying capacity(1, 2). The transmission rates and rapid emergence of novel strains are particularly challenging all conventional epidemiological wisdom and, by extension, effective deployment of routine contact tracing tools (3). At the peak of Uganda's first COVID-19 wave, community spread was significantly attributed to haulage drivers' interaction and seeding the infection in communities that live along the road network, particularly linked to service stations(4). This triggered the requirement to demonstrate proof of health; a negative COVID-19 PCR test result for all haulage drivers at Uganda's ports of entry and exit (POEs). This inevitably resulted in a pile-up that brought the entire regional supply chain to a halt. For example, in May 2020, Uganda had a queue of up to 47km of haulage trucks at Busia and Malaba border ports between Uganda-Kenya(5), which prompted the COVID-19 task force at the Ministry of Health, in consultation with the National Logistic Platform (de facto trade union for haulage drivers) to develop a mitigation plan to this problem(**Fig 1**).This plan aimed to limit haulage's risk of introducing the virus and variants into communities along the road network. Haulage drivers were allowed to obtain a single negative test result valid for 14 days. The success of this plan would have to be underwritten by a faster contact tracing approach for cases and their immediate contacts to support decision making and resource allocation (6)(**Fig 1**).

Effective and timely contact tracing slows the progression of community spread which addresses the economic and public health concerns that arise from mandatory lockdowns(7). In this regard, case investigation and faster contact tracing are considered primary mitigating interventions to control and prevent the person-to-person spread of COVID-19. This identifies and notifies persons with a confirmed diagnosis and their primary contacts.
Uganda has, over the years, developed contact tracing strategies for Ebola, Marburg and Crimean-Congo Haemorrhagic fever (CCHF) outbreaks(8). With such strategies, the surveillance teams were able to identify and isolate cases, in addition to their primary and secondary contacts. However, this "shoestring epidemiology" is too slow for fast-evolving epidemics and pandemics such as COVID-19.

To improve the speed and efficiency of conventional contact tracing (CCT), geolocation features integrated within mobile phone have been used to track proximity which can translate into contact once one becomes a case for a disease of interest(9). This digital contact tracing (DCT) approach automatically notifies individuals who test positive and their immediate contacts to test and/or isolate. It is worth noting population-wide uptake of such technology is incumbent on the level of smartphone ownership, possibly the current bottleneck for low-income countries like Uganda. Therefore targeted use becomes the most viable alternative, however, such technology is inherently public-facing as it harnesses information directly from mobile phones (10, 11). This inevitably comes with ethical, legal and sociological issues that must be robustly addressed to ensure sustainable adoption for public health. In a nutshell, DCT deployment requires a delicate balance between public health benefit, which is fairly distributed against the risk of harm and basic principles of ethics (12, 13).

In countries like the United States, various forms of this technology have been used to support contact tracing with varying levels of success. Although success depends on the scale of deployment, better outcomes have been reported with targeted use for private companies, particularly in Silicon Valley and sports franchises like American football(14, 15). Therefore countries intending to adopt this technology ought to examine and respond to the following evaluation questions; a) Can a DCT tool be effective in augmenting the current public health responses? b) If so, to what degree? c) Which specific aspects? d) With what confidence? and e) What are the prerequisites that ensure a DCT tool delivers on public health goals in a way that is ethically, legally and socially defensible?(16).

Here we target the haulage sector with the aim of showing how DCT can be developed and deployed to support public health needs in a way that meets the recommended sociological, ethical and legal boundaries in East Africa (17). We have started by developing a DCT tool prototype, with input from the relevant stakeholders; this will be further developed and tested in Uganda. (Link to download the current prototype; https://www.project-thea.org). It is noteworthy that our feedback will be collected through stakeholder meetings to be held throughout the project lifecycle (***Fig 2)***.

Our team will also examine the interaction between end users' (knowledge, attitudes, and practices) and societal context (bioethics and legal) regarding COVID-19 and how this influences technology uptake and ultimately impacts COVID-19 mitigation and infection control measures. We target the haulage sector based on the hypothesis that our digital contact tracing tool can harness their unique but predictable mobility using mobile phone technology embedded with; a) Road network infrastructure, b) time-stamped geo-positioning and c) COVID-19 test result data. Together these parameters will be optimised to increase the speed and accuracy of public health contact tracing in this region.

This will achieved through the following specific objectives:

1. To conduct stakeholder consultative meetings that inform the design of a haulage sector tailored-DCT .
2. To develop the DCT technology.
3. To test the utility of the DCT among truck drivers.
4. To develop models for mapping risk, transmission and estimate haulage attribution to COVID-19 epidemiology in Uganda.
5. To conduct stakeholder dialogue with feedback and review of potential benefits and harms of DCT technology.

Methods

Study Design

The COVID-19 pandemic presents a continuously changing epidemiological landscape; therefore, the study designs will be adaptable to accommodate unforeseen and unusually unpredictable circumstances. The project will generally use a mixture of methods tailored to each of the project components. For example, i) Stakeholder engagement will use qualitative and quantitative approaches to generate data. This will be done on a representative number along the hierarchy of the stakeholders in a cross-sectional study, ii) Develop a DCT tool with stakeholder feedback for contextualisation, iii) A longitudinal study to test the DCT tool with truck drivers will capture data using the mobile application.

Study setting

The study will primarily focus on the East African haulage network, particularly that which transits through Uganda (Fig 3A). Uganda has 52 ports of entry and exit (POEs), with varying levels of haulage traffic, categorised as; i) high, ii) moderate, iii) low-traffic POEs. This study will focus on one seclusion point in Kampala and five POEs, with the heaviest traffic routes with a combined haulage network traffic coverage of 45%.

Study participants

Identification and recruitment of key stakeholders- The key are local and regional haulage truck drivers. Other stakeholders include public health experts, including port-health and

surveillance departments of the Ministry of Health, the National Logistics Platform (NLP), Ministry of Transport, border point entry clearance and exit officials, legal, internet security and software developers, bioethicists as well as local leaders within neighbouring communities of service stations. There are approximately 123,000 registered haulage truck drivers in Uganda, 15% of whom are involved in regional haulage(18). Study participants will be recruited with guidance from the National Logistics Platform (NLP).

Sample size

Formative research study: We will recruit a diverse sample of truck drivers from any of the six study sites (**Fig 3A**). Other stakeholders will be purposively selected to ensure diverse experience and expertise. We estimate that a maximum of 30 participants will participate in three Focus Group Discussions (FGDs), with another 6-12 individuals taking part in the Key Informant Interviews (KII), while the self-administered questionnaire-based survey will generate data from approximately 300 respondents. The sample size estimation for examining knowledge attitudes and practices related to COVID-19 among haulage drivers and allied stakeholders will be done based on the methodology published by this group (16)

Quantitative study: We shall conduct a survey regarding stakeholder input on the mobile application's utility, functionality, and privacy. We will aim to recruit about 65% of the ~123,000 truck drivers to test the utility of the mobile application. This estimate is based on data from countries where this type of technology has been successfully tested, with a recommendation to target approximately 60-80% of the study population (18-20). If we target 65% of the study population, this will represent 11,200 (regional haulage drivers) and 72,000 (local haulage truck drivers), respectively(21). Our inclusion criteria will be a) consent to participate in the study and b), ownership of a smartphone that runs on Android or MAC IOS operating systems.

Procedures

First objective aims to conduct stakeholder consultative meetings to inform the design of the DCT tool tailored to the haulage sector in Uganda. To develop technology that embodies users' values, we shall conduct three stakeholder consultative meetings over the next 18 months. Using a stratified sampling method, we will identify a representative sample of key stakeholders such as regional and local haulage truck drivers, conductors and border point entry and exit clearance officials with varied characteristics that might modify the effects of the tool. These will include the individuals' age, experience, gender and education (***Fig 2***). It is noteworthy that we shall limit face to face interactions and use digital communication as much as possible, including hosting virtual structured discussions at regional and local entry and exit ports; and project meetings between partners.

Patient and public involvement
In addition to engaging key stakeholders as described above, we will engage research participants in developing and testing a mobile-based digital contact tracing (DCT) tool that incorporates their input for contextualisation.

The first workshop will focus on; a) mapping the knowledge, attitudes and practices (KAP) around COVID-19 among stakeholders in the haulage industry. This will be done using key informant interviews (KII), structured questionnaires, as well as break-away focus group discussions (FGD). b) In addition, we shall assemble a panel of communication experts to develop awareness and sensitization materials targeting the study population. We will focus on empowering participants with the knowledge needed to protect their ethical, social and legal boundaries with regards to technology, c) We shall also conduct a survey to collect stakeholder input on the functionality and privacy settings of our mobile application.

For peer review only

The second workshop will focus on informed consent, such as how one voluntarily joins or withdraws from the study, optimise parameters suitable to protect personal freedoms, and highlight in-built ethical and technology boundaries. It is noteworthy that the KAP survey will be done at all consultative meetings. The second focus group discussion will comprise of long-distance truck drivers. In contrast, the third will comprise of public health experts to maximise feedback on the functionality and utility of the mobile application. Approximately 30 participants will account for saturation with regard to focus group discussions (22), although each meeting will include only 10 participants at a time, lasting not more than an hour.

Second objective will be to develop a DCT tool to support COVID-19 surveillance. This process uses feedback from *objective one;* to inform the development of the DCT tool. We will employ a human-centred design approach- an iterative process of idea generation, prototyping, user-testing and piloting that involves end- users in the development process(23) (***Fig 3B***)(24**)**. The application will be developed to embody values that maximise user anonymity, flexibility, minimal data storage and battery life.

Third objective will be to test the utility of the DCT tool among haulage truck drivers. This will be achieved through truck drivers' interaction with the tool. Once the mobile application is downloaded and installed by the driver, it will; a) ask for consent from the driver to access the GPS b) request for information on regular haulage routes such as departure and destination. In this regard, the mobile app will have a feature that a user can choose to allow its activation whenever movement relative to the road network is detected i.e. "auto-activation when on road network". Finally, c) the application will then generate a universally unique identifier (UUID) or "digital token" as a pseudonym and deposit it into the secure, centralized database. Once processes (1) to (2) in (**Fig 4 & 5)** are completed, the app will launch and run in the background.

As an option, the app will also request the user for their metadata (age, sex, district of residence and phone contact), which will also be stored encrypted on the phone. The three pieces of information i.e. UUID, sampled GPS and metadata are kept encrypted separately. It is noteworthy that the regional haulage truck drivers will be required to have a negative test valid for 14 days, although a driver can cross the border 2-4 times a month (17).

COVID-19 testing: COVID-19 testing and reporting at POEs, as shown by steps 3 to 5 in (Fig 5), will be conducted by public health officials. Here we will collaborate with all Ministry of Health approved laboratories for testing truck drivers at POEs in Uganda, and these include: a) Test and Fly and b) Maia Medical laboratories in Kampala, Mpondwe, Mutukula, Malaba and Elegu. Before a sample is collected for the PCR test from the driver, consent will be sought, and then the THEA-C19 App will be installed on the smartphone. The App scans a QR-code of the East Africa truck driver's number used as laboratory sample ID. The laboratory will then post the results into the centralised results dispatch system (RDS) managed by the Ministry of Health. The scanned QR-codes will generate a list of ID's, which our system will use to retrieve results from the RDS. Here the DCT system will do the following:

1. Retrieve and notify the driver of their results.
2. If the result is positive, the driver will receive a notification and the current guidance by the ministry of health as a reminder.
3. Public health will then use the tracking data to create a 3-day risk map for cases which will be available as a web interface accessible to the public health surveillance team to support decision making.

When fully functional, the system can also inform risk-based screening, saving haulage companies money and time in testing.

Contact tracing: This will be implemented via the web interface only accessible to the Ministry of Health, Figure 4A. The interface will generate a contact graph from the time-stamped GPS

For peer review only - http://bmjopen.bmj.com/site/about/guidelines.xhtml

locations relative to the road infrastructure as shown in figure 4B and Step 5 (Fig 5) and overlay it with the COVID test results to reveal the risk trail cases in the last three days. For example, three people tested positive, as shown in figure 4C. The system will generate a list of all drivers, including the positive in the previous three days on the road network. Using a decision tree approach, checks where they stopped and for how long, and which stops overlap with the positive drivers. This will then generate a list of potentially exposed individuals which will then be ranked by the time of overlap. Drivers with the most extended time overlap at a given location will then be notified via the mobile application, considered as most likely exposed (see figure 4C)

Community risk assessment: Our system will generate a list of potentially exposed drivers for every positive test. These potentially exposed drivers represent the tool's prediction which in reality should be linked to a transmission event. The system will then compute the number of potentially exposed drivers on the list who test positive within the following 7-14 days to generate a "percentage predicted transmission (PPT)" metric. Since a transmission event is associated with a location linked to communities on the road network, over time, public health can use the PPT to predict/estimate the risk for the communities liked to the road network.

Fourth Objective will be to develop models for mapping risk, infer transmission and estimate a haulage attribution - The data collected by the mobile application (DCT) will be analysed as follows: Firstly, we will use machine learning methods to compute the probability of drivers testing positive as a function of characteristics of their movements (e.g. their route, duration of the journey, the departure time, vehicle type (~fuelling frequency) estimated traffic and contact structure. Determining a probabilistic output will enable the public health officials to prioritise who to test (risk-based diagnostics), limiting the number of contacts between infected drivers and individuals of the community. Note that since this process will need a training dataset, the first four months will be to generate this data set (~ 160,000 trips). Therefore, the utility of this approach for this analysis will be after six months of data collection.

Secondly the movements of trucks along the road networks will form a large network where nodes such as villages/towns/cities and edges are movements of haulage vehicles. This network will then be analysed to evaluate which communities are key in the structure of the network, their potential for disease transmission and infection as well as their potential as candidates for placing efficient testing facility outposts.

Lastly, assuming all drivers test negative at departure, we will estimate the likelihood of infection from communities along each individual travel route based on the spatial and temporal aspect of each individual journey, the time of testing, factoring in the epidemiological characteristics of COVID-19 (latent, incubation and symptomatic periods) in addition to probability of clinical signs. Based on this evidence, communities with a high risk of infection (either as source or recipient of infection) will be identified. Such a risk assessment would provide timely evidence for targeting communities with high risk of disease and improve resource allocation. By combining this with other data, we shall estimate the contribution of haulage to the epidemic and evaluate the impact of using this intervention on COVID-19 control, however the last component of the analysis will likely occur beyond the 18 months project period.

Fifth Objective is to conduct stakeholder dialogues to explore the impact of the mobile application (DCT) on disease control and preparedness. Deliberative dialogues are a form of public discourse that are currently used to develop a shared understanding of a problem or solution among key stakeholders(25). The objectives of these dialogues will "not so much to talk together as to think together, not so much to reach a conclusion as to discover where a conclusion might lie" and to ensure that different viewpoints and assumptions are explored(26). Dialogues also provide opportunities for tacit knowledge to be shared(27, 28).Using deliberative dialogues, we will create spaces where study findings are discussed

For peer review only

and explored by key stakeholders, provide opportunities to examine the extent to which the study findings are applicable to their contexts and how use of the mobile application technology could be formalized and scaled up to ensure appropriate governance and mechanisms to maintain data privacy. These stakeholder dialogues will follow the same approach as described for the KAP above.

Formative research data analysis- will be done using interpretative description approaches (29), such as those used in ethnography and naturalistic inquiry to analyse data arising from the KAP survey, KII and FGDs targeting the stakeholders. These approaches allow us to examine the practical implications of DCT on the routine activities of the truck drivers in the context of the existing body of knowledge in an iterative analysis, while exploring challenges and opportunities to using DCT technologies. We shall use qualitative analysis software, such as Nvivo to code and interrogate our data.

Data collection, protection and management

The team will take the following steps to secure the mobile app and THEA-C19 server:

1. Use transport layer security (TLS) for all communication between the mobile app and THEA C19 server.
2. Add rate limiting to the API service to prevent denial of service attacks and ensure availability at all times.
3. Maintain an access log for all system accesses.
4. Sanitize and validate all data submitted to the server to prevent cross-site scripting and SQL injection attacks.
5. Use the latest version of the frameworks, runtimes and database software to ensure the latest security patches are in place. Actively monitor CVE disclosures for all software packages used in order to respond to any reported vulnerabilities quickly.

There are extra security layers embedded which are not disclosed here to ensure the utility of the tools is tamperproof.

The contact tracing component will primarily use the collected time-stamped GPS data, which will be strictly limited to the road network, and are securely collected and transmitted to a server. The data collected will be stored in a relational database and will be retrievable in a number of formats including csv for analysis. The database will be replicated online to provide redundancy and ensure uninterrupted operation. In addition, daily backups are taken in the event that we need to restore the data which will be kept for the duration of the project. Use of the data, will only be triggered by a case; it is this case's risk profile that informs the contact tracing and notification via the mobile application. Otherwise all data will be kept separate. Diagnostic data will be generated and posted to a secure public health centralised system also referred to as the Result Dispatch System (RDS).

The stakeholder engagement component will generate qualitative data, collected as part of the Knowledge Attitude and Practices analysis linked to COVID-19, ethics and legal aspects of technology deployment. It will consist of audio recordings, observational notes, transcribed texts and still photography. Participants will be required to consent before downloading or installing the app. All participant data will be anonymised and stored securely on our servers for analysis. In addition, sequence data will be generated from a selected number of samples using Oxford Nano pore, this data once superimposed on the network, allows us to monitor the routes of COVID-19 strain introduction. The processing of this data will generate secondary data to be securely stored at the African Center for Excellence in Bio Informatics (ACE) at the Infectious Diseases Institute at Makerere University.

For peer review only

Project deliverables

1. *Open source Digital Contact Tracing Tool (DCT):*

We shall develop an open source Digital Contact Tracing (DCT) tool tailored to haulage in Uganda, to enhance the speed and efficiency of the current manual of public health contact tracing system. In so doing, we shall redirect resources from manual tracing, subsequently reducing border transit time for haulage while indirectly supporting increased supply chain flow.

2. *Statistical and mathematical models:*

Furthermore, the modelling team will develop statistical and mathematical models using output from testing for epidemic prediction and risk mapping. The outputs will provide evidence to inform, a) public health decision making on personnel and resource allocation critical to improving the efficacy, cost-efficiency and ultimately safety and surveillance of contact tracing activities, b) future control strategies to mitigate the spread of COVID-19 and other subsequent novel pathogens while limiting economic consequences and strengthening the resilience of the commodities' supply chain.

3. *Avail evidence for vaccination programs:*

The contact tracing analysis will provide epidemic characteristics, such as the speed of spatial transmission and size of the epidemic in specific locations, for example current data on hotspots. The data can then be used to define prioritisation of vaccination, estimate vaccination coverage while providing evidence on vaccination impact (i.e. temporal and spatial-changes) in the epidemic size.

4. *Avail evidence for policy:*

Lastly, any lessons learned regarding the legal, ethical and social-anthropological examination related to DCT deployment will be used to regulate usage and inform policy formulation in relation to use of digital contact tracing technology in combatting pandemics.

5. Interpretation and reporting of findings

Building on the deliberative dialogues, a small group of key stakeholders will be engaged in interpreting and confirming interpretations of findings. They will be invited to comment on reports and be co-authors.

Ethical considerations

In Uganda, ethics approval was obtained from School of Public Health Higher Degrees, Research and Ethics Committee at Makerere University (approval number SPH-2021-35) and Uganda National Council for Science and Technology (approval number HS156ES). At the University of Edinburgh, study approval was granted by the Human Ethics and Research Committee at the Easter Bush Campus (HERC_538).

We will obtain informed consent for participation in interviews, focus group discussions, still photography and videography. Data from interviews, questionnaires, GPS, photos, videos and notes will be anonymised.

Benefits of participation

Since clinically valid tests such as COVID-19 PCR test, are part of this protocol, research participants may benefit from individual results provided conveniently without financial cost. However, since research participants will not be provided any therapeutic interventions, consent forms clearly state that no financial benefits shall be gained as a result of individual participation in this research study, however haulage companies may ultimately benefit

For peer review only

monetarily by reducing their employees' exposure to the virus thereby limiting sickness-related absences while maintaining operations.

Risks of participation

In general, risks of participation resulting from both psychological and physical intervention necessary to acquire a biological sample are trivial. The primary risks to participation in this study are generally psychological, such as stigma and discrimination if sensitive information is generated in the project and there is a breach of privacy or confidentiality. However, industry standards that ensure confidentiality and privacy will be observed and maintained at all levels of data collection and processing to further mitigate that risk. No personal identification details will be linked to individuals as participants will be assigned unique identification pseudonyms, "digital tokens". The project will be conducted in accordance with the Ministry of Health and Uganda National Council for Science and Technology guidelines for the prevention of spread of COVID- 19.

Dissemination and reporting

We will provide all participating organisations with a report of the main findings as soon as the analyses have been completed and independently verified by at least two referees who are not part of the study team.

We will actively disseminate the results of this study through publications and presentations. At National level, the study is nested within the Ministry of Heath, with study outputs directly feeding into the public health responses via incident command teams. At the regional level, study findings will be presented at regional haulage sector meetings facilitated by the National Logistics Platform in Uganda. Internationally, we will actively disseminate the study findings through peer reviewed publications, pre-existing partnerships and our organisation websites such as https://project-thea.org/ and Github account for the open source code https://github.com/project-thea/

Data availability statement

The data files resulting from this study will be made available at reasonable request.

Funding

This study is funded by the National Institute of Health Research/ Medical Research Council-GECO award, ref MR/V034952/1

Author contributions

Conceptualisation: (Adrian Muwonge, Emmanuel Ssebaggala, Moses Joloba, Ibrahim Mugerwa)
Funding acquisition: (Adrian Muwonge, Christine Mpyangu, Ibrahim Mugerwa, Mark Bronsvoort[1], Thibaud Porphyre, Emmanuel Robert Ssebaggala, Aggelos Kiayias, Erisa Mwaka, Moses Joloba)
Investigation: Adrian Muwonge, Christine Mpyangu, Allen Nsangi, Ibrahim Mugerwa, Mark Bronsvoort[1], Thibaud Porphyre, Emmanuel Robert Ssebaggala, Aggelos Kiayias, Erisa Mwaka, Moses Joloba
Writing- original draft: (Adrian Muwonge, Christine Mpyangu, Allen Nsangi, Ibrahim Mugerwa, Mark Bronsvoort[1], Thibaud Porphyre, Emmanuel Robert Ssebaggala, Aggelos Kiayias, Erisa Mwaka, Moses Joloba)
Writing- review and editing: Adrian Muwonge, Christine Mpyangu, Allen Nsangi, Ibrahim Mugerwa, Mark Bronsvoort[1], Thibaud Porphyre, Emmanuel Robert Ssebaggala, Aggelos Kiayias, Erisa Mwaka, Moses Joloba

For peer review only

Declaration of interests

We declare no competing interests.

References

1. El-Sadr W, Justman J. Africa in the path of covid-19. N Engl J Med. 2020;383:e11.
2. Tumwesigye NM, Denis O, Kaakyo M, Biribawa C. Effects of the COVID-19 Pandemic on Health Services and Mitigation Measures in Uganda. Center for Global Development, 2021.
3. World Health Organization. Global research on coronavirus disease (COVID-19). World Health Organisation. 2021.
4. Ministry of Health. Coronavirus (COVID-19) Pandemic; COVID-19 Testing File Report- August 03 2020. Ministry of Health- Uganda. 2020.
5. Journal TWS. On East Africa's Borders, Vast Jams as Truckers Are Tested for Covid-19. The Wall Street Journal. 2020.
6. Roberts SL. Big Data, Algorithmic Governmentality and the Regulation of Pandemic Risk. European Journal of Risk Regulation,. 2019;10(1):94-115.
7. Haider N, Osman AY, Gadzekpo A, Akipede GO, Asogun D, Ansumana R, et al. Lockdown measures in response to COVID-19 in nine sub-Saharan African countries. BMJ Global Health. 2020;5(10):e003319.
8. Okware S. Managing Ebola in low-resource settings: experiences from Uganda. In Ebola IntechOpen
2016.
9. Blasimme A, Ferretti A, Vayena E. Digital Contact Tracing Against COVID-19 in Europe: Current Features and Ongoing Developments. Frontiers in Digital Health. 2021;3(61).
10. Mooney G. A Menace to the Public Health"—Contact Tracing and the Limits of Persuasion. New England Journal of Medicine. 2020.
11. Kahn JP. Digital contact tracing for pandemic response: Ethics and governance guidance. . John Hopkins University Press. 2020.
12. Bull S, Et al. Best practices for ethical sharing of individual-level health research data from low-and middle-income settings. . Journal of Empirical Research on Human Research Ethics. 2015;10(3):302-13.
13. Gasser U, Et al. Digital tools against COVID-19: taxonomy, ethical challenges, and navigation aid. Lancet Digital Health. 2020.
14. Kane C. How Kinexon— a German company with U.S. headquarters in Chicago — is tracking players' daily interactions to prevent the spread of COVID-19 in the NFL. 2020.
15. Center for Disease Control and Prevention. Digital contact tracing tools. 2020.
16. Muleme J, Kankya C, Ssempebwa JC, Mazeri S, Muwonge A. A Framework for Integrating Qualitative and Quantitative Data in Knowledge, Attitude, and Practice Studies: A Case Study of Pesticide Usage in Eastern Uganda. Frontiers in Public Health. 2017;5(318).
17. Keeling MJ, Hollingswworth TD, Read JM. The Efficacy of Contact Tracing for the Containment of the 2019 Novel Coronavirus (COVID-19). . medRxiv,. 2020.
18. BraithwaiteI I, Et al. Automated and partly automated contact tracing: a systematic review to inform the control of COVID-19. Lancet Digital Health. 2020.
19. Kim MS. Seoul's radical experiment in digital contact tracing 2020.
20. Rowe F. Contact tracing apps and values dilemmas: A privacy paradox in a neo-liberal world. International Journal of Information Management. 2020;55(102178).
21. Eberhard-Ruiz A, Calabrese L. Trade facilitation, transport costs and the price of trucking services in East Africa. Working and discussionpapers (August 2017) 2017.
22. Guest G BA, Johnson L. How many interviews Are enough?: anexperiment with data saturation and variability. Field Meth. 2006;2006(18):5-82.
23. Abras C, Maloney-Krichmar D, Preece J. User-Centered Design. In: Bainbridge W, editor. Encyclopedia of Human-Computer Interaction. Thousand Oaks:Sage Publi-cations, 2004;. 2004.
24. IDEO.org. The Field Guide to Human-Centered Design. . San Francisco: IDEOorg,. 2015;https://www.designkit.org/resources/1

25. The SURE Collaboration. Informing and engaging stakeholders. In: SURE Guides for Preparing and Using Evidence-Based Policy Briefs. Geneva: World Health Organization,. 2011;https://www.who.int/evidence/sure/guides/en/ 2011;Version 2.1(https://www.who.int/evidence/sure/guides/en/).
26. INVOLVE. People & Participation: How to put citizens at the heart of decision-making. London: Involve. 2005;https://www.involve.org.uk/resources/publications/practical-guidance/people-and-participation.
27. Hadjimichael D, Tsoukas H. Toward a better understanding of tacit knowledge in organizations: Taking stock and moving forward. Academy of Management Annals 2019;13(2):672-703.
28. Kothari A, Rudman D, Dobbins M, Et al. The use of tacit and explicit knowledge in public health: a qualitative study. Implementation Science. 2012;7(20).
29. Thorne S, Kirkham SR, O'Flynn-Magee K. The analytic challenge in interpretive description. Int J Qual Methods. 2004;3(1-11).

Figure legend

Figure 1: The study is responding to a public health and economic **challenge** linked to haulage sector as shown above. This group represents a **risk** of introducing COVID-19 and its variants to communities along the road infrastructure such as service stations, resting points and restaurants. This risk is exacerbated by delays which cause queuing up at POEs. Our **solution** is aimed at improving the efficiency of conventional contact tracing using **digital contact tracing**. The **utility**- is that this would allow for timely and robust **risk assessment**, automatic notification of primary and secondary contacts of identified cases as well as prediction of hotspots along the haulage network to support public health preparedness. The image was generated using Biorender https://biorender.com

Figure 2: Shows the conceptual framework on which this study is developed and will be implemented. The three interlocking wheels are used metaphorically to represent interlinkages between technology, individuals and communities. We posit that technology updake is enhanced by understanding inherent characteristics of the intended user (attitudes, knowledge and how these translate into actions) and the characteristics of the community they inhabit (legal, ethical, traditions and connectivity access). The project has four components; A is stakeholder engagement and inclusion, B- Technology development, C- Testing the technology and D -Data analysis to support public health interventions. The image was generated using Biorender https://biorender.com

Figure 3A-Shows the convergence of the East African haulage network in Uganda. The study will focus on the six ports of Entry and Exits (POEs) shown as circular highlights. **Figure 3**B Shows the iteration stages of the Human Centred Design Approach .The image was generated using Bio render https://biorender.com

Figure 4A Shows the architecture of the digital contact tracing tool how stakeholders feed or receive data on the system. **Figure 4B** show an example of the generated contact graph from the time-stamped GPS locations relative to the road infrastructure. In yellow, it highlights the hotspot where contact with an individual who tested positive occurred. **Figure 4C** shows the risk assessment process based on a decision tree; this identifies exposed individuals that the systems notify.

Figure 5: Demonstrates step by step the THEA-C19 mobile application installation and contact tracing processes

For peer review only

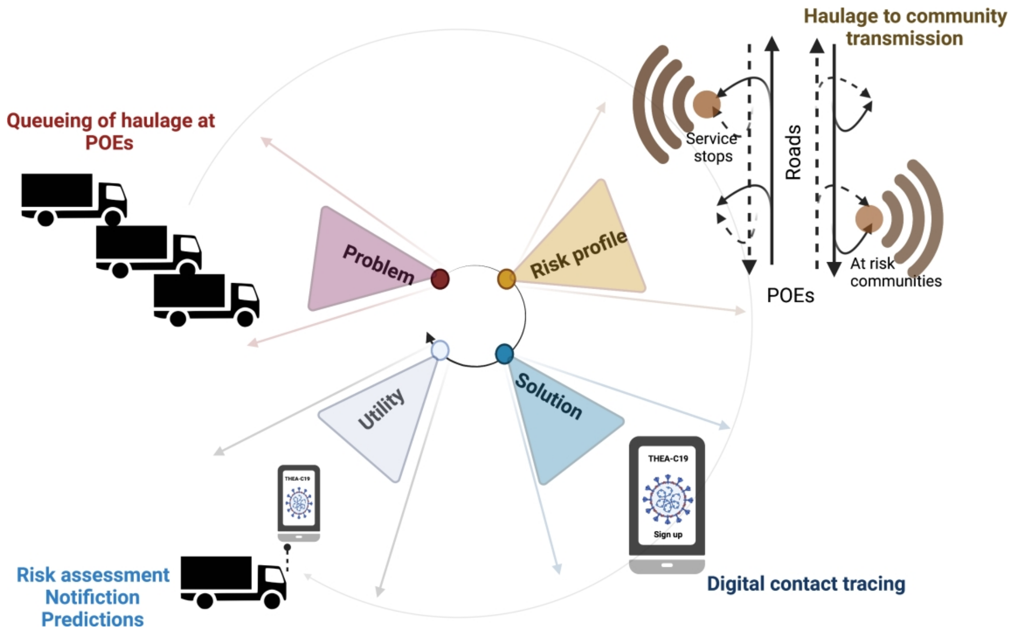

Figure 1: The study is responding to a public health and economic challenge linked to haulage sector as shown above. This group represents a risk of introducing COVID-19 and its variants to communities along the road infrastructure such as service stations, resting points and restaurants. This risk is exacerbated by delays which cause queuing up at POEs. Our solution is aimed at improving the efficiency of conventional contact tracing using digital contact tracing. The utility- is that this would allow for timely and robust risk assessment, automatic notification of primary and secondary contacts of identified cases as well as prediction of hotspots along the haulage network to support public health preparedness. The image was generated using Biorender https://biorender.com

249x154mm (301 x 301 DPI)

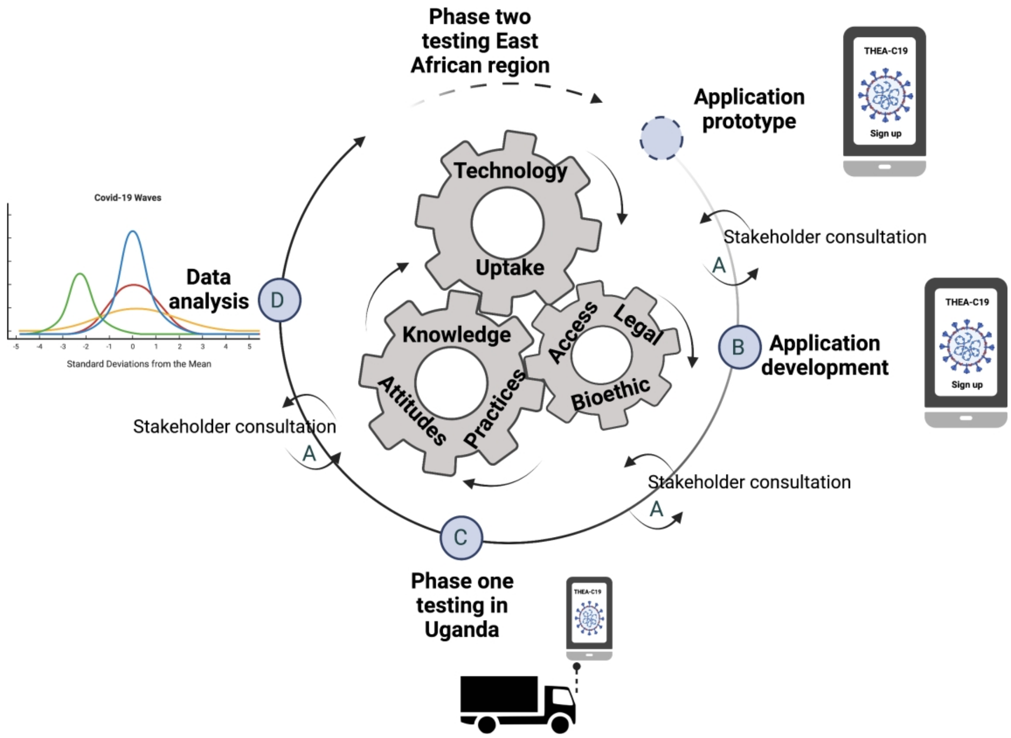

Figure 2: Shows the conceptual framework on which this study is developed and will be implemented. The three interlocking wheels are used metaphorically to represent interlinkages between technology, individuals and communities. We posit that technology updake is enhanced by understanding inherent characteristics of the intended user (attitudes, knowledge and how these translate into actions) and the characteristics of the community they inhabit (legal, ethical, traditions and connectivity access). The project has four components; A is stakeholder engagement and inclusion, B- Technology development, C- Testing the technology and D -Data analysis to support public health interventions. The image was generated using Biorender https://biorender.com

229x166mm (301 x 301 DPI)

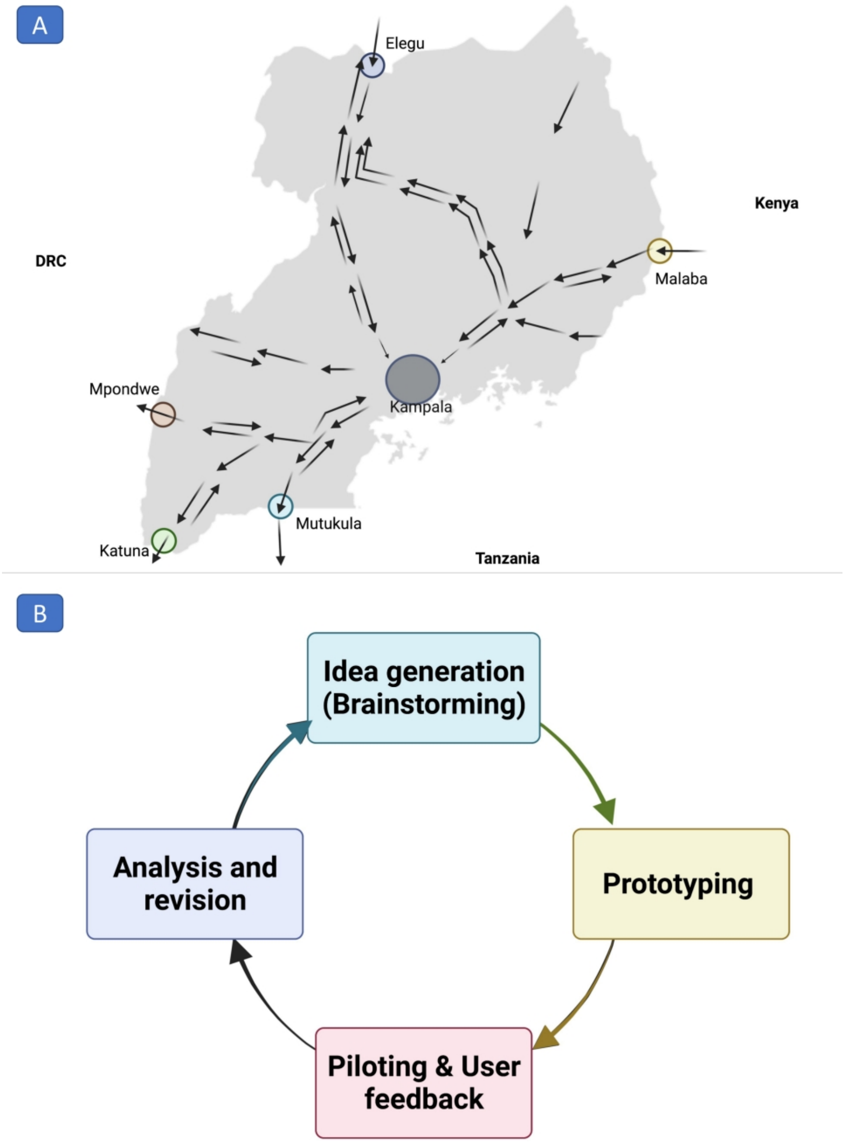

3A-Shows the convergence of the East African haulage network in Uganda. The study will focus on the six ports of Entry and Exits (POEs) shown as circular highlights. Figure 3B Shows the iteration stages of the Human Centred Design Approach .The image was generated using Bio render https://biorender.com

237x338mm (300 x 300 DPI)

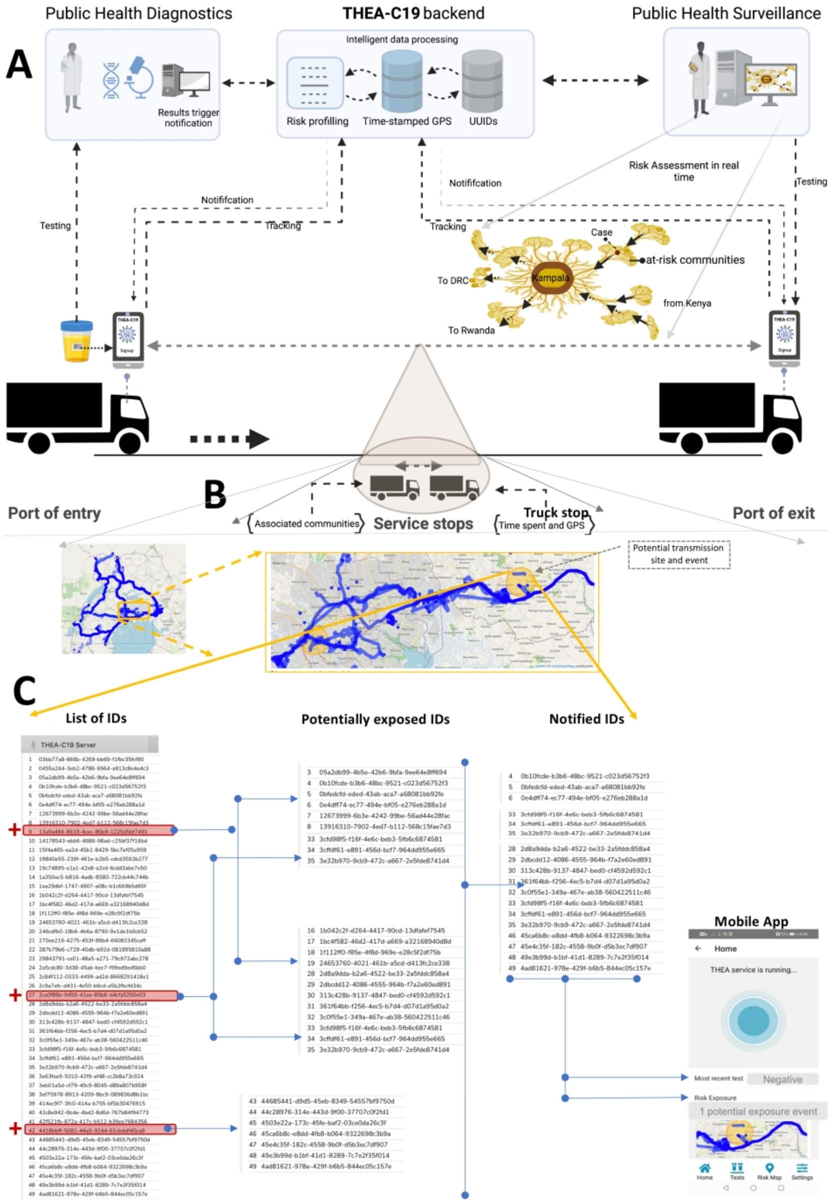

Shows the architecture of the digital contact tracing tool how stakeholders feed or receive data on the system. Figure 4B show an example of the generated contact graph from the time-stamped GPS locations relative to the road infrastructure. In yellow, it highlights the hotspot where contact with an individual who tested positive occurred. Figure 4C shows the risk assessment process based on a decision tree; this identifies exposed individuals that the systems notify.

234x338mm (300 x 300 DPI)

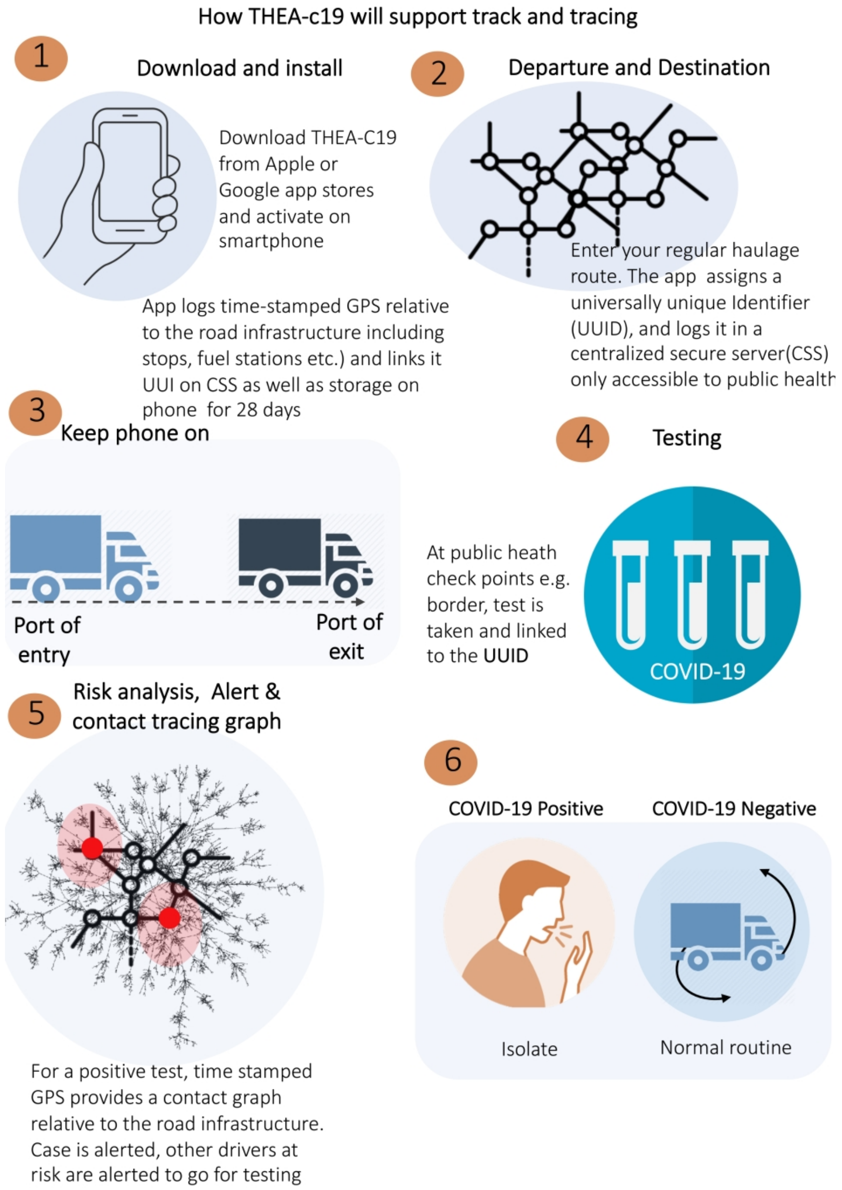

Figure 5: Demonstrates step by step the THEA-C19 mobile application installation and contact tracing processes

190x275mm (416 x 416 DPI)

CONSERVE Checklists

Use CONSERVE-CONSORT for completed trial reports and CONSERVE-SPIRIT for trial protocols.

CONSERVE-CONSORT Extension: [DATE]

**Item**	**Item Title**	**Description**	**Page No.**
I.	Extenuating Circumstances	Describe the circumstances and how they constitute extenuating circumstances.	
II.	Important Modifications	a. Describe how the modifications are important modifications.	
		b. Describe the impacts and mitigating strategies, including their rationale and implications for the trial.	(see below)
		c. Provide a modification timeline.	
III.	Responsible Parties	State who planned, reviewed and approved the modifications.	
IV.	Interim data	If modifications were informed by trial data, describe how the interim data were used, including whether they were examined by study group, and whether the individuals reviewing the data were blinded to the treatment allocation.	

**CONSORT Number and Item**		For each row, if important modifications occurred check "direct impact" and/or "mitigating strategy" and describe the changes in the trial manuscript or supplement. Check "no change" for items that are unaffected in the extenuating circumstance.			**Page No.**
		**No Change**	**Impact***	**Mitigating Strategy****	
1	Title and abstract				
2	Introduction				
3	Methods: Trial Design				
4	Methods: Participants				
5	Methods: Interventions				
6	Methods: Outcomes				

For peer review only

7	Methods: Sample Size				
8-10	Methods: Randomisation				
11	Methods: Blinding				
12	Methods: Statistical methods				
13	Results: Participant flow				
14	Results: Recruitment				
15	Results: Baseline data				
16	Results: Numbers analysed				
	Results: Outcomes and estimation				
18	Results: Ancillary analyses				
19	Results: Harms				
20	Discussion: Limitations				
21	Discussion: Generalisability				
	Other information: Registration				
24	Other information: Protocol				
25	Other information: Funding				

*Aspects of the trial that are directly affected or changed by the extenuating circumstance and are not under the control of investigators, sponsor or funder.
**Aspects of the trial that are modified by the study investigators, sponsor or funder to respond to the extenuating circumstance or manage the direct impacts on the trial.

**CONSERVE-SPIRIT Extension: [15th October 2021]**			
**Item**	**Item Title**	**Description**	**Page No.**
I.	Extenuating Circumstances	Describe the circumstances and how they constitute extenuating circumstances.	N/A
II.	Important Modifications	a. Describe how the modifications are important modifications.	N/A
		b. Describe the impacts and mitigating strategies, including their rationale and implications for the trial.	(see below)
		c. Provide a modification timeline.	N/A
III.	Responsible Parties	State who planned, reviewed and approved the modifications.	N/A
IV.	Interim data	If modifications were informed by trial data, describe how the interim data were used, including whether they were examined by study group, and whether the individuals reviewing the data were blinded to the treatment allocation.	N/A

**SPIRIT Item and Number**		For each row, if important modifications occurred, check one or both of "impact" and/or "mitigating strategy" and describe the changes in the protocol. Check "no change" for items that are unaffected in the extenuating circumstance.			**Page No.**
		**No Change**	**Impact***	**Mitigating Strategy****	
1	Title	√			1
2	Trial registration	√			N/A
3	Protocol version	√			N/A
4	Funding	√			11
5	Roles and responsibilities	√			11
6	Background and rationale	√			04
7	Objectives	√			05
8	Trial design	√			05
9	Study setting	√			05
10	Eligibility criteria	√			06
11	Interventions	√			05
12	Outcomes	√			09

For peer review only

13	Participant timeline	√			06
14	Sample size	√			06
15	Recruitment	√			06
16	Allocation	√			N/A
17	Blinding (masking)	√			N/A
18	Data collection methods	√			09
19	Data management	√			09
20	Statistical methods	√			09
21	Data monitoring	√			09
22	Harms	√			10
23	Auditing	√			N/A
24	Research ethics approval	√			10
25	Protocol amendments	√			N/A
26	Consent or assent	√			06
27	Confidentiality	√			10
28	Declaration of interests	√			11
29	Access to data	√			11
30	Ancillary and post-trial care	√			N/A
31	Dissemination policy	√			11
32	Informed consent materials	√			10
33	Biological specimens	√			N/A

*Aspects of the trial that are directly affected or changed by the extenuating circumstance and are not under the control of investigators, sponsor or funder.
**Aspects of the trial that are modified by the study investigators, sponsor or funder to respond to the extenuating circumstance or manage the direct impacts on the trial.

For peer review only

BMJ Open

A protocol for developing digital contact tracing tailored to haulage in East Africa to support COVID-19 surveillance

Journal:	*BMJ Open*
Manuscript ID	bmjopen-2021-058457.R2
Article Type:	Protocol
Date Submitted by the Author:	11-Aug-2022
Complete List of Authors:	Muwonge, Adrian; The University of Edinburgh The Roslin Institute, The Roslin Institute<br>Mpyangu, Christine; Makerere University College of Humanities and Social Sciences<br>Nsangi, Allen; Makerere University College of Health Sciences, Department of Medicine; Universitetet i Oslo, Institute of Health and Society, Faculty of Medicine<br>Mugerwa, Ibrahim; Ministry of Health, National Health Laboratories and Diagnostic Services, Antimicrobial Resistance National Coordination Centre (AMR-NCC) for<br>Bronsvoort, Barend M. deC.; The University of Edinburgh The Roslin Institute<br>Porphyre, Thibaud; VetAgro Sup, 89 Avenue de l'Europe, 63370 Lempdes<br>Ssebaggala, Emmanuel; Bodastage solution Limited<br>Kiayias, Aggelos; The University of Edinburgh School of Informatics, Blockchain Technology Laboratory<br>Mwaka, Erisa; Makerere University College of Health Sciences, School of Medicine<br>Joloba, Moses; Makerere University College of Health Sciences
**Primary Subject Heading**:	Public health
Secondary Subject Heading:	Epidemiology, Evidence based practice, Global health, Infectious diseases
Keywords:	COVID-19, Epidemiology < TROPICAL MEDICINE, Anthropology < TROPICAL MEDICINE, Public health < INFECTIOUS DISEASES

SCHOLARONE™
Manuscripts

A protocol for developing digital contact tracing tailored to haulage in East Africa to support COVID-19 surveillance

Adrian Muwonge[1,2]#, Christine Mpyangu[3], Allen Nsangi[4], Ibrahim Mugerwa[4], Barend M. deC. Bronsvoort[1], Thibaud Porphyre[1,6], Emmanuel Robert Ssebaggala[7], Aggelos Kiayias[2], Mwaka, Erisa Sabakaki [4], Moses Joloba[4]

The Roslin Institute, College of Medicine and Veterinary Medicine, University of Edinburgh, Scotland, United Kingdom[1], The Block chain Laboratory, School of Informatics, University of Edinburgh, Scotland, United Kingdom[2] , Makerere University College of Humanities and Social Sciences, Kampala, Uganda[3], Makerere University College of Health Sciences, Kampala, Uganda[4], Ministry of Health, Kampala, Uganda[5], VetAgro Sup, Lyon, France[6], Bodastage Solutions, Kampala, Uganda[7],

Corresponding Author:
Dr. Adrian Muwonge
Chancellor's Fellow (Digital One-Health)
University of Edinburgh, (U.K)
Address: The Roslin Institute, Easter Bush Campus, University of Edinburgh, Midlothian, EH25 9RG, United Kingdom)
Telephone: (01316519155)
Email: Adrian.muwonge@roslin.ed.ac.uk

For peer review only

Abstract

Introduction: At the peak of Uganda's first wave of SARS-Cov2 in May 2020, one in three COVID-19 cases was linked to the haulage sector. This triggered a mandatory requirement for a negative PCR test result at all ports of entry and exit (POEs), resulting in significant delays as haulage drivers had to wait for 24-48 hours for results, which severely crippled the regional supply chain.

To support public health and economic recovery, we aim to develop and test a mobile phone-based digital contact tracing (DCT) tool that not only augments conventional contact tracing but increases its speed and efficiency.

Methods and analysis: To test the DCT tool, we will use a stratified sample of haulage driver journeys, stratified by route type (regional and local journeys).

We will include at least 65% of the haulage driver journeys ~ 83,200 on the network through Uganda. This allows us to capture variations in user demographics and socio-economic characteristics that could influence the use and adoption of the DCT tool. The developed DCT tool will include a mobile application and web interface to collate and intelligently process data, whose output will support decision making, resource allocation and feed mathematical models that predict epidemic waves.

The main expected result will be an open source tested Digital Contact Tracing (DCT) Tool tailored to haulage use in developing countries.

This study will inform the safe deployment of digital contact tracing technologies needed for combatting pandemics in low-income countries.

Ethics and dissemination: This work has received ethics approval from School of Public Health Higher Degrees, Research and Ethics Committee at Makerere University and The Uganda National Council for Science and Technology. This work will be disseminated through peer reviewed publications, our websites https://project-thea.org/ and Github for the open source code https://github.com/project-thea/

Key words: *Digital contact-tracing, haulage, COVID-19, Uganda, East Africa*

For peer review only

Strengths and limitations of the study

Strengths

- This is the first study that fully documents the development of a DCT tool in consultation with stakeholders on the African continent.
- It brings together a multidisciplinary team to provide a solution to an old and neglected problem “the role of haulage in transmission of infectious diseases”. Therefore, although the proposed DCT tool is linked to the COVID-19 pandemic, its utility goes far beyond the current pandemic.

Weaknesses

- The ever-changing epidemiological landscape of COVID-19, means the study design is subject to adjustments to accommodate the prevailing public health demands. These will however be openly highlighted and discussed in the resulting publications.

For peer review only

Background

The Corona Virus Disease of 2019 (COVID-19), caused by the SARS-CoV2 virus, is a global pandemic that has stretched public health systems worldwide, often beyond carrying capacity(1, 2). The transmission rates and rapid emergence of novel strains are particularly challenging all conventional epidemiological wisdom and, by extension, effective deployment of routine contact tracing tools (3). At the peak of Uganda's first COVID-19 wave, community spread was significantly attributed to haulage drivers' interaction and seeding the infection in communities that live along the road network, particularly linked to service stations(4). This triggered the requirement to demonstrate proof of health; a negative COVID-19 PCR test result for all haulage drivers at Uganda's ports of entry and exit (POEs). This inevitably resulted in a pile-up that brought the entire regional supply chain to a halt. For example, in May 2020, Uganda had a queue of up to 47km of haulage trucks at Busia and Malaba border ports between Uganda-Kenya(5), which prompted the COVID-19 task force at the Ministry of Health, in consultation with the National Logistic Platform (de facto trade union for haulage drivers) to develop a mitigation plan to this problem(**Fig 1**).This plan aimed to limit haulage's risk of introducing the virus and variants into communities along the road network. Haulage drivers were allowed to obtain a single negative test result valid for 14 days. The success of this plan would have to be underwritten by a faster contact tracing approach for cases and their immediate contacts to support decision making and resource allocation (6)(**Fig 1**).

Effective and timely contact tracing slows the progression of community spread which addresses the economic and public health concerns that arise from mandatory lockdowns(7). In this regard, case investigation and faster contact tracing are considered primary mitigating interventions to control and prevent the person-to-person spread of COVID-19. This identifies and notifies persons with a confirmed diagnosis and their primary contacts.
Uganda has, over the years, developed contact tracing strategies for Ebola, Marburg and Crimean-Congo Haemorrhagic fever (CCHF) outbreaks(8). With such strategies, the surveillance teams were able to identify and isolate cases, in addition to their primary and secondary contacts. However, this "shoestring epidemiology" is too slow for fast-evolving epidemics and pandemics such as COVID-19.

To improve the speed and efficiency of conventional contact tracing (CCT), geolocation features integrated within mobile phone have been used to track proximity which can translate into contact once one becomes a case for a disease of interest(9). This digital contact tracing (DCT) approach automatically notifies individuals who test positive and their immediate contacts to test and/or isolate. It is worth noting population-wide uptake of such technology is incumbent on the level of smartphone ownership, possibly the current bottleneck for low-income countries like Uganda. Therefore targeted use becomes the most viable alternative, however, such technology is inherently public-facing as it harnesses information directly from mobile phones (10, 11). This inevitably comes with ethical, legal and sociological issues that must be robustly addressed to ensure sustainable adoption for public health. In a nutshell, DCT deployment requires a delicate balance between public health benefit, which is fairly distributed against the risk of harm and basic principles of ethics (12, 13).

In countries like the United States, various forms of this technology have been used to support contact tracing with varying levels of success. Although success depends on the scale of deployment, better outcomes have been reported with targeted use for private companies, particularly in Silicon Valley and sports franchises like American football(14, 15). Therefore countries intending to adopt this technology ought to examine and respond to the following evaluation questions; a) Can a DCT tool be effective in augmenting the current public health responses? b) If so, to what degree? c) Which specific aspects? d) With what confidence? and e) What are the prerequisites that ensure a DCT tool delivers on public health goals in a way that is ethically, legally and socially defensible?(16).

For peer review only

Here we target the haulage sector with the aim of showing how DCT can be developed and deployed to support public health needs in a way that meets the recommended sociological, ethical and legal boundaries in East Africa (17). We have started by developing a DCT tool prototype, with input from the relevant stakeholders; this will be further developed and tested in Uganda. (Link to download the current prototype; (https://www.project-thea.org). It is noteworthy that our feedback will be collected through stakeholder meetings to be held throughout the project lifecycle (***Fig 2)***.

Our team will also examine the interaction between end users' (knowledge, attitudes, and practices) and societal context (bioethics and legal) regarding COVID-19 and how this influences technology uptake and ultimately impacts COVID-19 mitigation and infection control measures. We target the haulage sector based on the hypothesis that our digital contact tracing tool can harness their unique but predictable mobility using mobile phone technology embedded with; a) Road network infrastructure, b) time-stamped geo-positioning and c) COVID-19 test result data. Together these parameters will be optimised to increase the speed and accuracy of public health contact tracing in this region.

This will be achieved through the following specific objectives:

1. To conduct stakeholder consultative meetings that inform the design of a haulage sector tailored-DCT.
2. To develop the DCT technology.
3. To test the utility of the DCT among truck drivers.
4. To develop models for mapping risk, transmission and estimate haulage attribution to COVID-19 epidemiology in Uganda.
5. To conduct stakeholder dialogue with feedback and review of potential benefits and harms of DCT technology.

Methods

Study Design

The COVID-19 pandemic presents a continuously changing epidemiological landscape; therefore, the study designs will be adaptable to accommodate unforeseen and unusually unpredictable circumstances. The project will generally use a mixture of methods tailored to each of the project components. For example, i) Stakeholder engagement will use qualitative and quantitative approaches to generate data. This will be done on a representative number along the hierarchy of the stakeholders in a cross-sectional study, ii) Develop a DCT tool with stakeholder feedback for contextualisation, iii) A longitudinal study to test the DCT tool with truck drivers will capture data using the mobile application.

Study setting

The field study will start in January 2022 for one year, primarily focusing on the East African haulage network, particularly that which transits through Uganda (Fig 3A). Uganda has 52 ports of entry and exit (POEs), with varying levels of haulage traffic, categorised as; i) high, ii) moderate, iii) low-traffic POEs. This study will focus on one seclusion point in Kampala and five POEs, with the heaviest traffic routes with a combined haulage network traffic coverage of 45%.

For peer review only

Study participants

Identification and recruitment of key stakeholders- The key are local and regional haulage truck drivers. Other stakeholders include public health experts, including port-health and surveillance departments of the Ministry of Health, the National Logistics Platform (NLP), Ministry of Transport, border point entry clearance and exit officials, legal, internet security and software developers, bioethicists as well as local leaders within neighbouring communities of service stations. There are approximately 123,000 registered haulage truck drivers in Uganda, 15% of whom are involved in regional haulage(18). Study participants will be recruited with guidance from the National Logistics Platform (NLP).

Sample size

Formative research study: We will recruit a diverse sample of truck drivers from any of the six study sites (**Fig 3A**). Other stakeholders will be purposively selected to ensure diverse experience and expertise. We estimate that a maximum of 30 participants will participate in three Focus Group Discussions (FGDs), with another 6-12 individuals taking part in the Key Informant Interviews (KII), while the self-administered questionnaire-based survey will generate data from approximately 300 respondents. The sample size estimation for examining knowledge attitudes and practices related to COVID-19 among haulage drivers and allied stakeholders will be done based on the methodology published by this group (16)

Quantitative study: We shall conduct a survey regarding stakeholder input on the mobile application's utility, functionality, and privacy. We will aim to recruit about 65% of the ~123,000 truck drivers to test the utility of the mobile application. This estimate is based on data from countries where this type of technology has been successfully tested, with a recommendation to target approximately 60-80% of the study population (18-20). If we target 65% of the study population, this will represent 11,200 (regional haulage drivers) and 72,000 (local haulage truck drivers), respectively(21). Our inclusion criteria will be a) consent to participate in the study and b), ownership of a smartphone that runs on Android or MAC IOS operating systems.

Procedures

First objective aims to conduct stakeholder consultative meetings to inform the design of the DCT tool tailored to the haulage sector in Uganda. To develop technology that embodies users' values, we shall conduct three stakeholder consultative meetings over the next 18 months. Using a stratified sampling method, we will identify a representative sample of key stakeholders such as regional and local haulage truck drivers, conductors and border point entry and exit clearance officials with varied characteristics that might modify the effects of the tool. These will include the individuals' age, experience, gender and education (***Fig 2***). It is noteworthy that we shall limit face to face interactions and use digital communication as much as possible, including hosting virtual structured discussions at regional and local entry and exit ports; and project meetings between partners.

Patient and public involvement

In addition to engaging key stakeholders as described above, we will engage research participants in developing and testing a mobile-based digital contact tracing (DCT) tool that incorporates their input for contextualisation.

The first workshop will focus on; a) mapping the knowledge, attitudes and practices (KAP) around COVID-19 among stakeholders in the haulage industry. This will be done using key informant interviews (KII), structured questionnaires, as well as break-away focus group discussions (FGD). b) In addition, we shall assemble a panel of communication experts to develop awareness and sensitization materials targeting the study population. We will focus

For peer review only - http://bmjopen.bmj.com/site/about/guidelines.xhtml

on empowering participants with the knowledge needed to protect their ethical, social and legal boundaries with regards to technology, c) We shall also conduct a survey to collect stakeholder input on the functionality and privacy settings of our mobile application.

The second workshop will focus on informed consent, such as how one voluntarily joins or withdraws from the study, optimise parameters suitable to protect personal freedoms, and highlight in-built ethical and technology boundaries. It is noteworthy that the KAP survey will be done at all consultative meetings. The second focus group discussion will comprise of long-distance truck drivers. In contrast, the third will comprise of public health experts to maximise feedback on the functionality and utility of the mobile application. Approximately 30 participants will account for saturation with regard to focus group discussions (22), although each meeting will include only 10 participants at a time, lasting not more than an hour.

Second objective will be to develop a DCT tool to support COVID-19 surveillance. This process uses feedback from *objective one;* to inform the development of the DCT tool. We will employ a human-centred design approach- an iterative process of idea generation, prototyping, user-testing and piloting that involves end- users in the development process(23) **(*Fig 3B*)**(24**)**. The application will be developed to embody values that maximise user anonymity, flexibility, and minimal data storage and battery life.

Third objective will be to test the utility of the DCT tool among haulage truck drivers. This will be achieved through truck drivers' interaction with the tool. Once the mobile application is downloaded and installed by the driver, it will; a) ask for consent from the driver to access the GPS b) request for information on regular haulage routes such as departure and destination. In this regard, the mobile app will have a feature that a user can choose to allow its activation whenever movement relative to the road network is detected i.e. "auto-activation when on road network". Finally, c) the application will then generate a universally unique identifier (UUID) or "digital token" as a pseudonym and deposit it into the secure, centralized database. Once processes (1) to (2) in (**Fig 4 & 5)** are completed, the app will launch and run in the background.

As an option, the app will also request the user for their metadata (age, sex, district of residence and phone contact), which will also be stored encrypted on the phone. The three pieces of information i.e. UUID, sampled GPS and metadata are kept encrypted separately. It is noteworthy that the regional haulage truck drivers will be required to have a negative test valid for 14 days, although a driver can cross the border 2-4 times a month (17).

COVID-19 testing: COVID-19 testing and reporting at POEs, as shown by steps 3 to 5 in (Fig 5), will be conducted by public health officials. Here we will collaborate with all Ministry of Health approved laboratories for testing truck drivers at POEs in Uganda, and these include: a) Test and Fly and b) Maia Medical laboratories in Kampala, Mpondwe, Mutukula, Malaba and Elegu. Before a sample is collected for the PCR test from the driver, consent will be sought, and then the THEA-C19 App will be installed on the smartphone. The App scans a QR-code of the East Africa truck driver's number used as laboratory sample ID. The laboratory will then post the results into the centralised results dispatch system (RDS) managed by the Ministry of Health. The scanned QR-codes will generate a list of ID's, which our system will use to retrieve results from the RDS Here the DCT system will do the following:

1. Retrieve and notify the driver of their results.
2. If the result is positive, the driver will receive a notification and the current guidance by the ministry of health as a reminder.
3. Public health will then use the tracking data to create a 3-day risk map for cases which will be available as a web interface accessible to the public health surveillance team to support decision making.

4. Once notified, the case and contacts are required to quarantine based on the guideline, adherence will be assess using the inherent geo-fencing on road network. For example; if a notified case or their contact are detected on the road network within ten days from notification date, our system will flag this as an event of non-adherence subject to further investigation by a public health official.

When fully functional, the system can generate time logs for events and their notifications, and this data allows us to evaluate the overall speed of the digital contact tracing process. We can also identify time lags between critical steps; for example, on average, it takes one hour for a test result to be posted in the result dispatch system (RDS) of the ministry of health. The tool can then calculate the time from result posting to case notification, risk assessment and contact notification. For technical reasons, the THEA- server will query the RDS every 30 minutes to retrieve new results; this means result notification could take up to one hour and a half from sample collection and contact notification up to one hour after that. The frequency of notification will be optimised for prudent computing capacity allocation for risk assessment.
Notably, the output can inform future risk-based screening, which would save haulage companies money and time in testing.

Contact tracing: This will be implemented via the web interface only accessible to the Ministry of Health, Figure 4A. The interface will generate a contact graph from the time-stamped GPS locations relative to the road infrastructure as shown in figure 4B and Step 5 (Fig 5) and overlay it with the COVID test results to reveal the risk trail cases in the last three days. For example, three people tested positive, as shown in figure 4C. The system will generate a list of all drivers, including the positive in the previous three days on the road network. Using a decision tree approach, checks where they stopped and for how long, and which stops overlap with the positive drivers. This will then generate a list of potentially exposed individuals which will then be ranked by the time of overlap. Drivers with the most extended time overlap at a given location will then be notified via the mobile application, considered as most likely exposed (see figure 4C).

Community risk assessment: Our system will generate a list of potentially exposed drivers for every positive test. These potentially exposed drivers represent the tool's prediction which in reality should be linked to a transmission event. The system will then compute the number of potentially exposed drivers on the list who test positive within the following 7-14 days to generate a "percentage predicted transmission (PPT)" metric. Since a transmission event is associated with a location linked to communities on the road network, over time, public health can use the PPT to predict/estimate the risk for the communities liked to the road network. Notably, we estimate that asymptomatic and pre-symptomatic cases will account for 15% and 52% of the notified positive cases, respectively(25), which are associated with a lower secondary transmission risk. Such estimates will be contextualised using our empirical data and used to evaluate the tool’s effectiveness for contact tracing, i.e. the sensitivity and proportion of expected false positive contact notification.
Fourth Objective will be to develop models for mapping risk, infer transmission and estimate a haulage attribution - The data collected by the mobile application (DCT) will be analysed as follows: Firstly, we will use machine learning methods to compute the probability of drivers testing positive as a function of characteristics of their movements (e.g. their route, duration of the journey, the departure time, vehicle type (~fuelling frequency) estimated traffic and contact structure. Determining a probabilistic output will enable the public health officials to prioritise who to test (risk-based diagnostics), limiting the number of contacts between infected drivers and individuals of the community. Note that since this process will need a training dataset, the first four months will be to generate this data set (~ 160,000 trips). Therefore, the utility of this approach for this analysis will be after six months of data collection.

Secondly the movements of trucks along the road networks will form a large network where nodes such as villages/towns/cities and edges are movements of haulage vehicles. This

For peer review only

network will then be analysed to evaluate which communities are key in the structure of the network, their potential for disease transmission and infection as well as their potential as candidates for placing efficient testing facility outposts.

Lastly, assuming all drivers test negative at departure, we will estimate the likelihood of infection from communities along each individual travel route based on the spatial and temporal aspect of each individual journey, the time of testing, factoring in the epidemiological characteristics of COVID-19 (latent, incubation and symptomatic periods) in addition to probability of clinical signs. Based on this evidence, communities with a high risk of infection (either as source or recipient of infection) will be identified. Such a risk assessment would provide timely evidence for targeting communities with high risk of disease and improve resource allocation. By combining this with other data, we shall estimate the contribution of haulage to the epidemic and evaluate the impact of using this intervention on COVID-19 control, however the last component of the analysis will likely occur beyond the 18 months project period.

Fifth Objective is to conduct stakeholder dialogues to explore the impact of the mobile application (DCT) on disease control and preparedness. Deliberative dialogues are a form of public discourse that are currently used to develop a shared understanding of a problem or solution among key stakeholders(26). The objectives of these dialogues will "not so much to talk together as to think together, not so much to reach a conclusion as to discover where a conclusion might lie" and to ensure that different viewpoints and assumptions are explored(27). Dialogues also provide opportunities for tacit knowledge to be shared(28, 29).Using deliberative dialogues, we will create spaces where study findings are discussed and explored by key stakeholders, provide opportunities to examine the extent to which the study findings are applicable to their contexts and how use of the mobile application technology could be formalized and scaled up to ensure appropriate governance and mechanisms to maintain data privacy. These stakeholder dialogues will follow the same approach as described for the KAP above.

Formative research data analysis- will be done using interpretative description approaches (30), such as those used in ethnography and naturalistic inquiry to analyse data arising from the KAP survey, KII and FGDs targeting the stakeholders. These approaches allow us to examine the practical implications of DCT on the routine activities of the truck drivers in the context of the existing body of knowledge in an iterative analysis, while exploring challenges and opportunities to using DCT technologies. We shall use qualitative analysis software, such as Nvivo to code and interrogate our data.

Data collection, protection and management

The team will take the following steps to secure the mobile app and THEA-C19 server:

1. Use transport layer security (TLS) for all communication between the mobile app and THEA C19 server.
2. Add rate limiting to the API service to prevent denial of service attacks and ensure availability at all times.
3. Maintain an access log for all system accesses.
4. Sanitize and validate all data submitted to the server to prevent cross-site scripting and SQL injection attacks.
5. Use the latest version of the frameworks, runtimes and database software to ensure the latest security patches are in place. Actively monitor CVE disclosures for all software packages used in order to respond to any reported vulnerabilities quickly.

There are extra security layers embedded which are not disclosed here to ensure the utility of the tools is tamperproof.

For peer review only

The contact tracing component will primarily use the collected time-stamped GPS data, which will be strictly limited to the road network, and are securely collected and transmitted to a server. The data collected will be stored in a relational database and will be retrievable in a number of formats including csv for analysis. The database will be replicated online to provide redundancy and ensure uninterrupted operation. In addition, daily backups are taken in the event that we need to restore the data which will be kept for the duration of the project. Use of the data, will only be triggered by a case; it is this case's risk profile that informs the contact tracing and notification via the mobile application. Otherwise all data will be kept separate. Diagnostic data will be generated and posted to a secure public health centralised system also referred to as the Result Dispatch System (RDS).

The stakeholder engagement component will generate qualitative data, collected as part of the Knowledge Attitude and Practices analysis linked to COVID-19, ethics and legal aspects of technology deployment. It will consist of audio recordings, observational notes, transcribed texts and still photography. Participants will be required to consent before downloading or installing the app. All participant data will be anonymised and stored securely on our servers for analysis. In addition, sequence data will be generated from a selected number of samples using Oxford Nano pore, this data once superimposed on the network, allows us to monitor the routes of COVID-19 strain introduction. The processing of this data will generate secondary data to be securely stored at the African Center for Excellence in Bio Informatics (ACE) at the Infectious Diseases Institute at Makerere University.

Project deliverables

1. *Open source Digital Contact Tracing Tool (DCT):*

We shall develop an open source Digital Contact Tracing (DCT) tool tailored to haulage in Uganda, to enhance the speed and efficiency of the current manual of public health contact tracing system. In so doing, we shall redirect resources from manual tracing, subsequently reducing border transit time for haulage while indirectly supporting increased supply chain flow.

2. *Statistical and mathematical models:*

Furthermore, the modelling team will develop statistical and mathematical models using output from testing for epidemic prediction and risk mapping. The outputs will provide evidence to inform, a) public health decision making on personnel and resource allocation critical to improving the efficacy, cost-efficiency and ultimately safety and surveillance of contact tracing activities, b) future control strategies to mitigate the spread of COVID-19 and other subsequent novel pathogens while limiting economic consequences and strengthening the resilience of the commodities' supply chain.

3. *Avail evidence for vaccination programs:*

The contact tracing analysis will provide epidemic characteristics, such as the speed of spatial transmission and size of the epidemic in specific locations, for example current data on hotspots. The data can then be used to define prioritisation of vaccination, estimate vaccination coverage while providing evidence on vaccination impact (i.e. temporal and spatial-changes) in the epidemic size.

4. *Avail evidence for policy:*

Lastly, any lessons learned regarding the legal, ethical and social-anthropological examination related to DCT deployment will be used to regulate usage and inform policy formulation in relation to use of digital contact tracing technology in combatting pandemics.

For peer review only

5. Interpretation and reporting of findings

Building on the deliberative dialogues, a small group of key stakeholders will be engaged in interpreting and confirming interpretations of findings. They will be invited to comment on reports and be co-authors.

The development of our digital contact trace tool is complete, and we are currently conducting a field study to test the functionality. In addition, our teams are currently conducting the ethical, social and legal dimensions of deploying such a tool for public health in the Kampala district. The project is on track to deliver on the aspects listed above.

Ethics and dissemination

In Uganda, ethics approval was obtained from School of Public Health Higher Degrees, Research and Ethics Committee at Makerere University (approval number SPH-2021-35) and Uganda National Council for Science and Technology (approval number HS156ES). At the University of Edinburgh, study approval was granted by the Human Ethics and Research Committee at the Easter Bush Campus (HERC_538).

We will obtain informed consent for participation in interviews, focus group discussions, still photography and videography. Data from interviews, questionnaires, GPS, photos, videos and notes will be anonymised.

Benefits of participation

Since clinically valid tests such as COVID-19 PCR test, are part of this protocol, research participants may benefit from individual results provided conveniently without financial cost. However, since research participants will not be provided any therapeutic interventions, consent forms clearly state that no financial benefits shall be gained as a result of individual participation in this research study, however haulage companies may ultimately benefit monetarily by reducing their employees' exposure to the virus thereby limiting sickness-related absences while maintaining operations.

Risks of participation

In general, risks of participation resulting from both psychological and physical intervention necessary to acquire a biological sample are trivial. The primary risks to participation in this study are generally psychological, such as stigma and discrimination if sensitive information is generated in the project and there is a breach of privacy or confidentiality. However, industry standards that ensure confidentiality and privacy will be observed and maintained at all levels of data collection and processing to further mitigate that risk. No personal identification details will be linked to individuals as participants will be assigned unique identification pseudonyms, "digital tokens". The project will be conducted in accordance with the Ministry of Health and Uganda National Council for Science and Technology guidelines for the prevention of spread of COVID- 19.

We will provide all participating organisations with a report of the main findings as soon as the analyses have been completed and independently verified by at least two referees who are not part of the study team.

We will actively disseminate the results of this study through publications and presentations. At National level, the study is nested within the Ministry of Heath, with study outputs directly feeding into the public health responses via incident command teams. At the regional level, study findings will be presented at regional haulage sector meetings facilitated by the National Logistics Platform in Uganda. Internationally, we will actively disseminate the study findings through peer reviewed publications, pre-existing partnerships and our organisation websites

For peer review only

such as https://project-thea.org/ and Github account for the open source code https://github.com/project-thea/

Data availability statement

The data files resulting from this study will be made available at reasonable request.

Funding

This study is funded by the National Institute of Health Research/ Medical Research Council-GECO award, ref MR/V034952/1

Author contributions

Conceptualisation: (Adrian Muwonge, Emmanuel Ssebaggala, Moses Joloba, Ibrahim Mugerwa)
Funding acquisition: (Adrian Muwonge, Christine Mpyangu, Ibrahim Mugerwa, Mark Bronsvoort[1], Thibaud Porphyre, Emmanuel Robert Ssebaggala, Aggelos Kiayias, Erisa Mwaka, Moses Joloba)
Investigation: Adrian Muwonge, Christine Mpyangu, Allen Nsangi, Ibrahim Mugerwa, Mark Bronsvoort[1], Thibaud Porphyre, Emmanuel Robert Ssebaggala, Aggelos Kiayias, Erisa Mwaka, Moses Joloba
Writing- original draft: (Adrian Muwonge, Christine Mpyangu, Allen Nsangi, Ibrahim Mugerwa, Mark Bronsvoort[1], Thibaud Porphyre, Emmanuel Robert Ssebaggala, Aggelos Kiayias, Erisa Mwaka, Moses Joloba)
Writing- review and editing: Adrian Muwonge, Christine Mpyangu, Allen Nsangi, Ibrahim Mugerwa, Mark Bronsvoort[1], Thibaud Porphyre, Emmanuel Robert Ssebaggala, Aggelos Kiayias, Erisa Mwaka, Moses Joloba

Declaration of interests

We declare no competing interests.

References

1. El-Sadr W, Justman J. Africa in the path of covid-19. N Engl J Med. 2020;383:e11.
2. Tumwesigye NM, Denis O, Kaakyo M, Biribawa C. Effects of the COVID-19 Pandemic on Health Services and Mitigation Measures in Uganda. Center for Global Development, 2021.
3. World Health Organization. Global research on coronavirus disease (COVID-19). World Health Organisation. 2021.
4. Ministry of Health. Coronavirus (COVID-19) Pandemic; COVID-19 Testing File Report-August 03 2020. Ministry of Health- Uganda. 2020.
5. Journal TWS. On East Africa's Borders, Vast Jams as Truckers Are Tested for Covid-19. The Wall Street Journal. 2020.
6. Roberts SL. Big Data, Algorithmic Governmentality and the Regulation of Pandemic Risk. European Journalof Risk Regulation,. 2019;10(1):94-115.
7. Haider N, Osman AY, Gadzekpo A, Akipede GO, Asogun D, Ansumana R, et al. Lockdown measures in response to COVID-19 in nine sub-Saharan African countries. BMJ Global Health. 2020;5(10):e003319.
8. Okware S. Managing Ebola in low-resource settings: experiences from Uganda. In Ebola IntechOpen 2016.
9. Blasimme A, Ferretti A, Vayena E. Digital Contact Tracing Against COVID-19 in Europe: Current Features and Ongoing Developments. Frontiers in Digital Health. 2021;3(61).

For peer review only

10. Mooney G. A Menace to the Public Health"—Contact Tracing and the Limits of Persuasion. New England Journal of Medicine. 2020.

11. Kahn JP. Digital contact tracing for pandemic response: Ethics and governance guidance. . John Hopkins University Press. 2020.

12. Bull S, Et al. Best practices for ethical sharing of individual-level health research data from low-and middle-income settings. . Journal of Empirical Research on Human Research Ethics. 2015;10(3):302-13.

13. Gasser U, Et al. Digital tools against COVID-19: taxonomy, ethical challenges, and navigation aid. Lancet Digital Health. 2020.

14. Kane C. How Kinexon — a German company with U.S. headquarters in Chicago — is tracking players' daily interactions to prevent the spread of COVID-19 in the NFL. 2020.

15. Center for Disease Control and Prevention. Digital contact tracing tools. 2020.

16. Muleme J, Kankya C, Ssempebwa JC, Mazeri S, Muwonge A. A Framework for Integrating Qualitative and Quantitative Data in Knowledge, Attitude, and Practice Studies: A Case Study of Pesticide Usage in Eastern Uganda. Frontiers in Public Health. 2017;5(318).

17. Keeling MJ, Hollingswworth TD, Read JM. The Efficacy of Contact Tracing for the Containment of the 2019 Novel Coronavirus (COVID-19). . medRxiv,. 2020.

18. Braithwaitel I, Et al. Automated and partly automated contact tracing: a systematic review to inform the control of COVID-19. Lancet Digital Health. 2020.

19. Kim MS. Seoul's radical experiment in digital contact tracing 2020.

20. Rowe F. Contact tracing apps and values dilemmas: A privacy paradox in a neo-liberal world. International Journal of Information Management. 2020;55(102178).

21. Eberhard-Ruiz A, Calabrese L. Trade facilitation, transport costs and the price of trucking services in East Africa. Working and discussionpapers (August 2017) 2017.

22. Guest G BA, Johnson L. How many interviews Are enough?: anexperiment with data saturation and variability. Field Meth. 2006;2006(18):5-82.

23. Abras C, Maloney-Krichmar D, Preece J. User-Centered Design. In: Bainbridge W, editor. Encyclopedia of Human-Computer Interaction. Thousand Oaks:Sage Publi-cations, 2004;. 2004.

24. IDEO.org. The Field Guide to Human-Centered Design. . San Francisco: IDEOorg,. 2015;https://www.designkit.org/resources/1

25. Gao W, Lv J, Pang Y, Li L-M. Role of asymptomatic and pre-symptomatic infections in covid-19 pandemic. BMJ. 2021;375:n2342.

26. The SURE Collaboration. Informing and engaging stakeholders. In: SURE Guides for Preparing and Using Evidence-Based Policy Briefs. Geneva: World Health Organization,. 2011;https://www.who.int/evidence/sure/guides/en/ 2011;Version 2.1(https://www.who.int/evidence/sure/guides/en/).

27. INVOLVE. People & Participation: How to put citizens at the heart of decision-making. London: Involve. 2005;https://www.involve.org.uk/resources/publications/practical-guidance/people-and-participation.

28. Hadjimichael D, Tsoukas H. Toward a better understanding of tacit knowledge in organizations: Taking stock and moving forward. Academy of Management Annals 2019;13(2):672-703.

29. Kothari A, Rudman D, Dobbins M, Et al. The use of tacit and explicit knowledge in public health: a qualitative study. Implementation Science. 2012;7(20).

For peer review only

30. Thorne S, Kirkham SR, O'Flynn-Magee K. The analytic challenge in interpretive description. Int J Qual Methods. 2004;3(1-11).

Figure legend

Figure 1: The study is responding to a public health and economic **challenge** linked to haulage sector as shown above. This group represents a **risk** of introducing COVID-19 and its variants to communities along the road infrastructure such as service stations, resting points and restaurants. This risk is exacerbated by delays which cause queuing up at POEs. Our **solution** is aimed at improving the efficiency of conventional contact tracing using **digital contact tracing**. The **utility-** is that this would allow for timely and robust **risk assessment**, automatic notification of primary and secondary contacts of identified cases as well as prediction of hotspots along the haulage network to support public health preparedness. The image was generated using Biorender https://biorender.com

Figure 2: Shows the conceptual framework on which this study is developed and will be implemented. The three interlocking wheels are used metaphorically to represent interlinkages between technology, individuals and communities. We posit that technology updake is enhanced by understanding inherent characteristics of the intended user (attitudes, knowledge and how these translate into actions) and the characteristics of the community they inhabit (legal, ethical, traditions and connectivity access). The project has four components; A is stakeholder engagement and inclusion, B- Technology development, C- Testing the technology and D -Data analysis to support public health interventions. The image was generated using Biorender https://biorender.com

Figure 3A; shows the convergence of the East African haulage network in Uganda. The study will focus on the six ports of Entry and Exits (POEs) shown as circular highlights. **Figure 3B**; shows the iteration stages of the Human Centred Design Approach .The image was generated using Bio render https://biorender.com

Figure 4A; shows the architecture of the digital contact tracing tool how stakeholders feed or receive data on the system. **Figure 4B** shows an example of the generated contact graph from the time-stamped GPS locations relative to the road infrastructure. In yellow, it highlights the hotspot where contact with an individual who tested positive occurred. **Figure 4C** shows the risk assessment process based on a decision tree; this identifies exposed individuals that the systems notify.

Figure 5: Demonstrates step by step the THEA-C19 mobile application installation and contact tracing processes

For peer review only

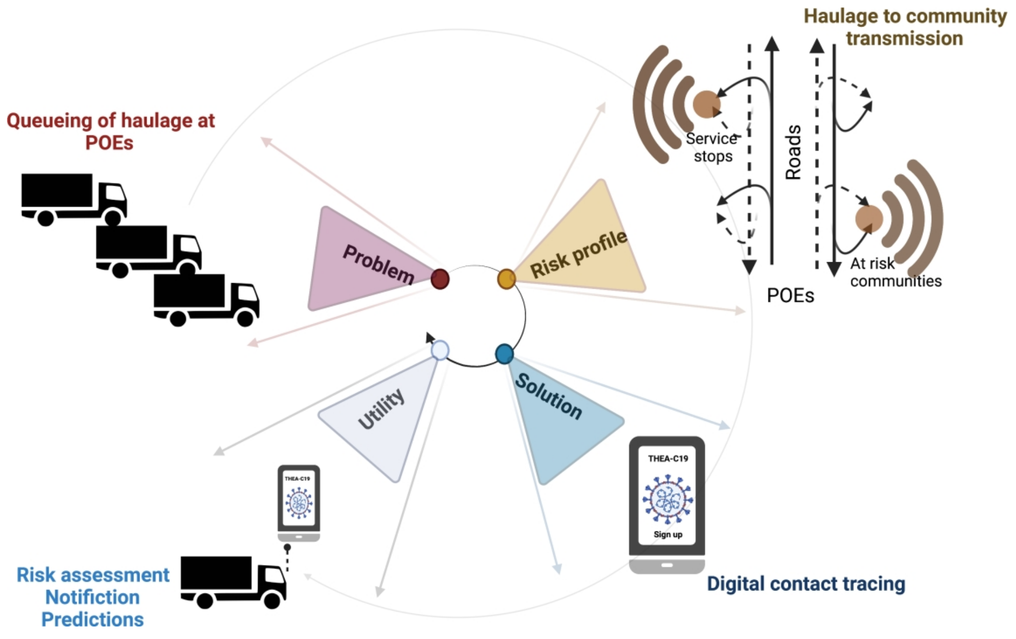

Figure 1: The study is responding to a public health and economic challenge linked to haulage sector as shown above. This group represents a risk of introducing COVID-19 and its variants to communities along the road infrastructure such as service stations, resting points and restaurants. This risk is exacerbated by delays which cause queuing up at POEs. Our solution is aimed at improving the efficiency of conventional contact tracing using digital contact tracing. The utility- is that this would allow for timely and robust risk assessment, automatic notification of primary and secondary contacts of identified cases as well as prediction of hotspots along the haulage network to support public health preparedness. The image was generated using Biorender https://biorender.com

249x154mm (301 x 301 DPI)

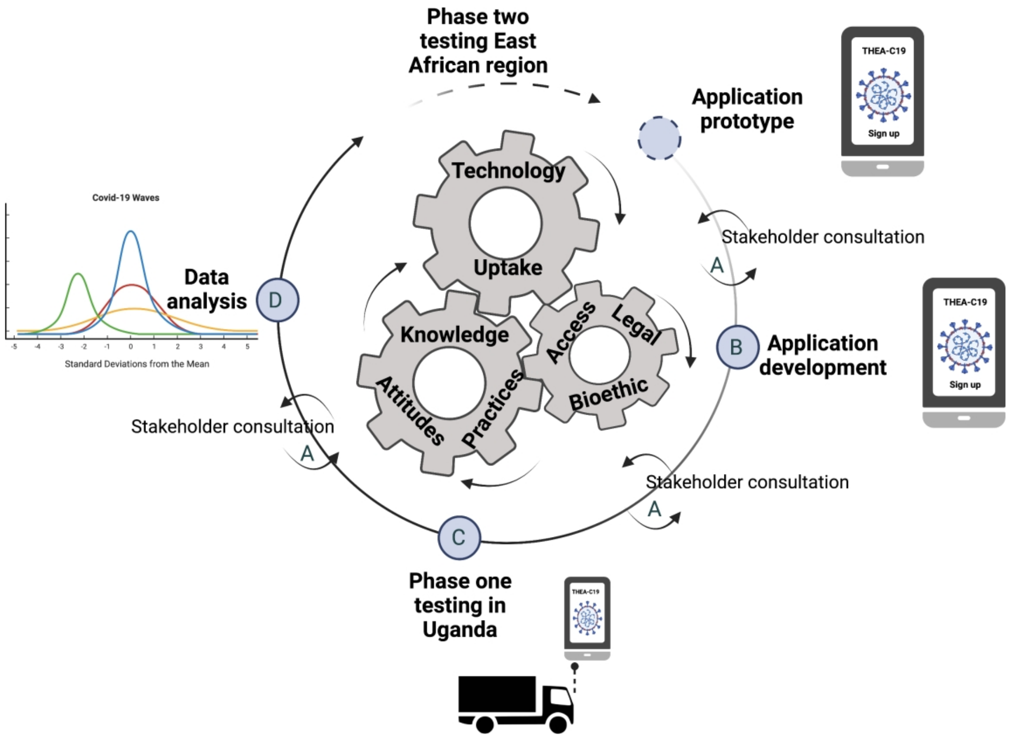

Figure 2: Shows the conceptual framework on which this study is developed and will be implemented. The three interlocking wheels are used metaphorically to represent interlinkages between technology, individuals and communities. We posit that technology updake is enhanced by understanding inherent characteristics of the intended user (attitudes, knowledge and how these translate into actions) and the characteristics of the community they inhabit (legal, ethical, traditions and connectivity access). The project has four components; A is stakeholder engagement and inclusion, B- Technology development, C- Testing the technology and D -Data analysis to support public health interventions. The image was generated using Biorender https://biorender.com

229x166mm (301 x 301 DPI)

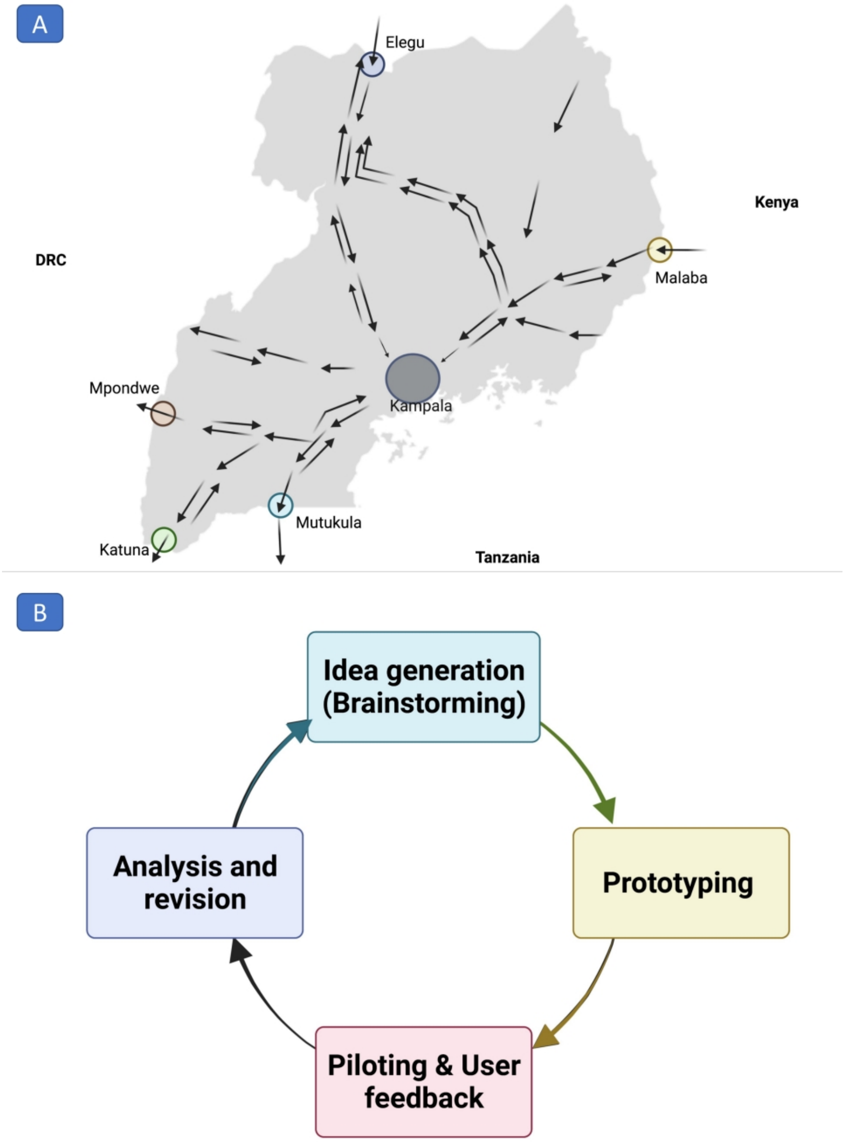

3A-Shows the convergence of the East African haulage network in Uganda. The study will focus on the six ports of Entry and Exits (POEs) shown as circular highlights. Figure 3B Shows the iteration stages of the Human Centred Design Approach .The image was generated using Bio render https://biorender.com

237x338mm (301 x 301 DPI)

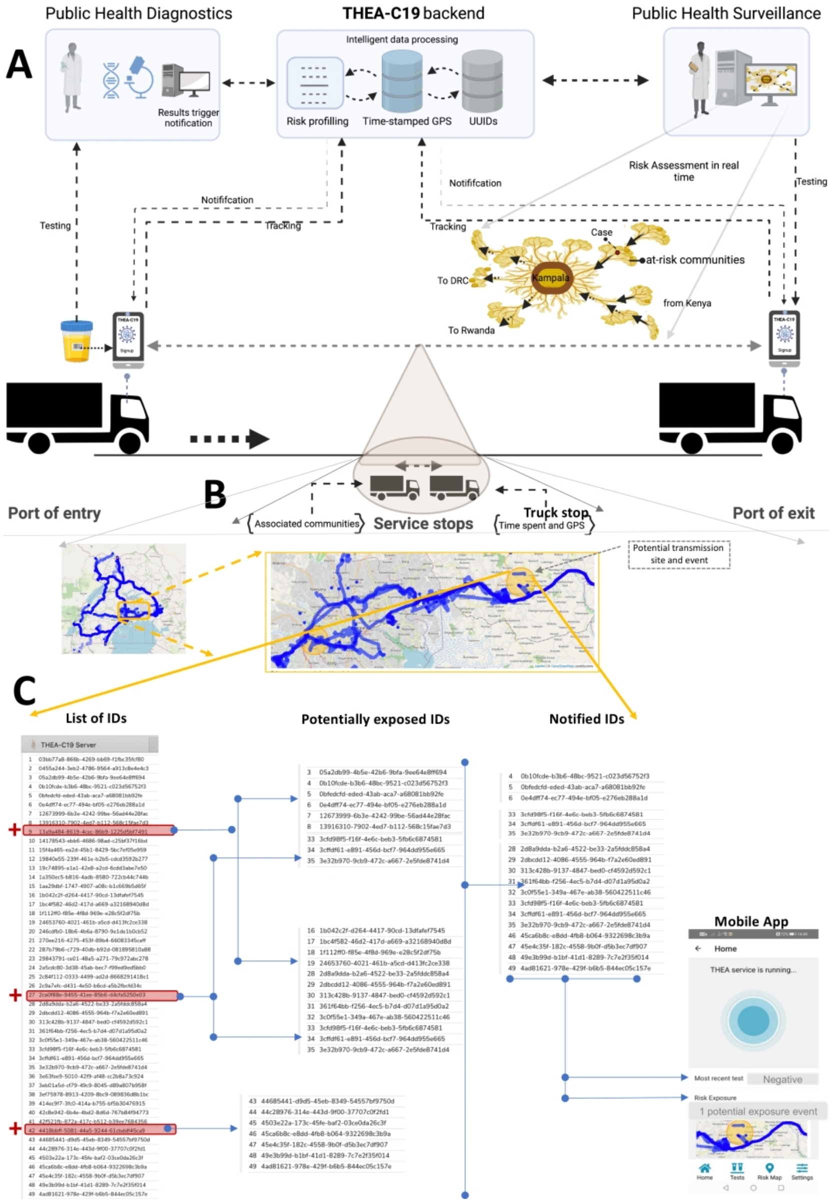

Shows the architecture of the digital contact tracing tool how stakeholders feed or receive data on the system. Figure 4B show an example of the generated contact graph from the time-stamped GPS locations relative to the road infrastructure. In yellow, it highlights the hotspot where contact with an individual who tested positive occurred. Figure 4C shows the risk assessment process based on a decision tree; this identifies exposed individuals that the systems notify.

234x338mm (350 x 350 DPI)

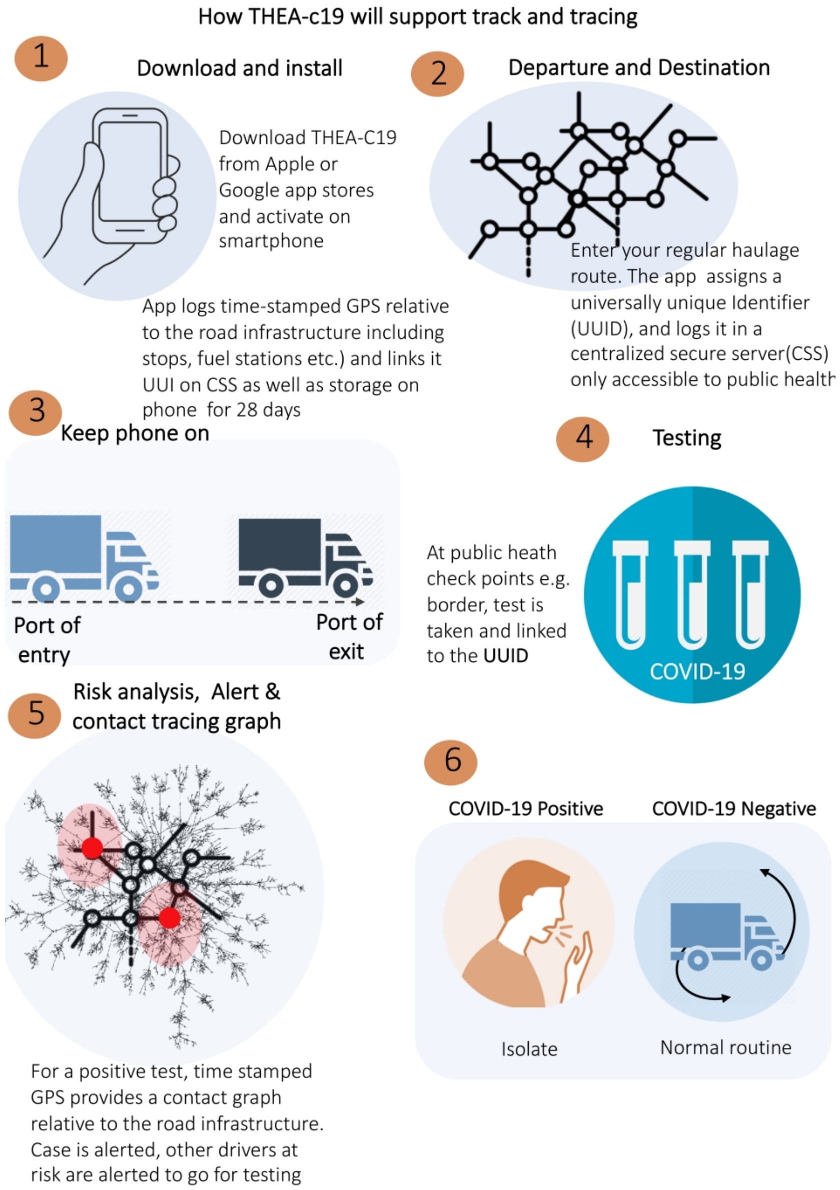

Figure 5: Demonstrates step by step the THEA-C19 mobile application installation and contact tracing processes

190x275mm (416 x 416 DPI)

CONSERVE Checklists

Use CONSERVE-CONSORT for completed trial reports and CONSERVE-SPIRIT for trial protocols.

**CONSERVE-CONSORT Extension: [DATE]**			
**Item**	**Item Title**	**Description**	**Page No.**
I.	Extenuating Circumstances	Describe the circumstances and how they constitute extenuating circumstances.	
II.	Important Modifications	a. Describe how the modifications are important modifications.	
		b. Describe the impacts and mitigating strategies, including their rationale and implications for the trial.	(see below)
		c. Provide a modification timeline.	
III.	Responsible Parties	State who planned, reviewed and approved the modifications.	
IV.	Interim data	If modifications were informed by trial data, describe how the interim data were used, including whether they were examined by study group, and whether the individuals reviewing the data were blinded to the treatment allocation.	

**CONSORT Number and Item**		For each row, if important modifications occurred check "direct impact" and/or "mitigating strategy" and describe the changes in the trial manuscript or supplement. Check "no change" for items that are unaffected in the extenuating circumstance.			**Page No.**
		**No Change**	**Impact***	**Mitigating Strategy****	
1	Title and abstract				
2	Introduction				
3	Methods: Trial Design				
4	Methods: Participants				
5	Methods: Interventions				
6	Methods: Outcomes				

For peer review only

For peer review only - http://bmjopen.bmj.com/site/about/guidelines.xhtml

7	Methods: Sample Size				
8-10	Methods: Randomisation				
11	Methods: Blinding				
12	Methods: Statistical methods				
13	Results: Participant flow				
14	Results: Recruitment				
15	Results: Baseline data				
16	Results: Numbers analysed				
	Results: Outcomes and estimation				
18	Results: Ancillary analyses				
19	Results: Harms				
20	Discussion: Limitations				
21	Discussion: Generalisability				
	Other information: Registration				
24	Other information: Protocol				
25	Other information: Funding				

*Aspects of the trial that are directly affected or changed by the extenuating circumstance and are not under the control of investigators, sponsor or funder.
**Aspects of the trial that are modified by the study investigators, sponsor or funder to respond to the extenuating circumstance or manage the direct impacts on the trial.

CONSERVE-SPIRIT Extension: [15th October 2021]

Item	Item Title	Description	Page No.
I.	Extenuating Circumstances	Describe the circumstances and how they constitute extenuating circumstances.	N/A
II.	Important Modifications	a. Describe how the modifications are important modifications.	N/A
		b. Describe the impacts and mitigating strategies, including their rationale and implications for the trial.	(see below)
		c. Provide a modification timeline.	N/A
III.	Responsible Parties	State who planned, reviewed and approved the modifications.	N/A
IV.	Interim data	If modifications were informed by trial data, describe how the interim data were used, including whether they were examined by study group, and whether the individuals reviewing the data were blinded to the treatment allocation.	N/A

SPIRIT Item and Number		For each row, if important modifications occurred, check one or both of "impact" and/or "mitigating strategy" and describe the changes in the protocol. Check "no change" for items that are unaffected in the extenuating circumstance.			Page No.
		**No Change**	**Impact***	**Mitigating Strategy****	
1	Title	√			1
2	Trial registration	√			N/A
3	Protocol version	√			N/A
4	Funding	√			11
5	Roles and responsibilities	√			11
6	Background and rationale	√			04
7	Objectives	√			05
8	Trial design	√			05
9	Study setting	√			05
10	Eligibility criteria	√			06
11	Interventions	√			05
12	Outcomes	√			09

For peer review only

13	Participant timeline	√			06
14	Sample size	√			06
15	Recruitment	√			06
16	Allocation	√			N/A
17	Blinding (masking)	√			N/A
18	Data collection methods	√			09
19	Data management	√			09
20	Statistical methods	√			09
21	Data monitoring	√			09
22	Harms	√			10
23	Auditing	√			N/A
24	Research ethics approval	√			10
25	Protocol amendments	√			N/A
26	Consent or assent	√			06
27	Confidentiality	√			10
28	Declaration of interests	√			11
29	Access to data	√			11
30	Ancillary and post-trial care	√			N/A
31	Dissemination policy	√			11
32	Informed consent materials	√			10
33	Biological specimens	√			N/A

*Aspects of the trial that are directly affected or changed by the extenuating circumstance and are not under the control of investigators, sponsor or funder.
**Aspects of the trial that are modified by the study investigators, sponsor or funder to respond to the extenuating circumstance or manage the direct impacts on the trial.

For peer review only
